# Tsukushi proteoglycan maintains RNA splicing and developmental signaling network in GFAP-expressing subventricular zone neural stem/progenitor cells

**DOI:** 10.3389/fcell.2022.994588

**Published:** 2022-11-21

**Authors:** Arif Istiaq, Terumasa Umemoto, Naofumi Ito, Toshio Suda, Kenji Shimamura, Kunimasa Ohta

**Affiliations:** ^1^ Department of Stem Cell Biology, Faculty of Arts and Science, Kyushu University, Fukuoka, Japan; ^2^ Department of Brain Morphogenesis, Institute of Molecular Embryology and Genetics, Kumamoto University, Kumamoto, Japan; ^3^ HIGO Program, Kumamoto University, Kumamoto, Japan; ^4^ International Research Center for Medical Sciences, Kumamoto University, Kumamoto, Japan; ^5^ Department of Stem Cell Biology, Graduate School of Systems Life Sciences, Kyushu University, Fukuoka, Japan

**Keywords:** tsukushi, hydrocephalus, neural stem cells, missplicing, lateral ventricles, GFAP-expressing cell

## Abstract

Tsukushi (TSK) proteoglycan dysfunction leads to hydrocephalus, a condition defined by excessive fluid collection in the ventricles and lateral ventricular enlargement. TSK injections into the LV at birth are effective at rescuing the lateral ventricle (LV). TSK regulates the activation of the Wnt signaling to facilitate the proper expansion of the LV and maintain the fate of the neural stem cell lineage. However, the molecular mechanism by which TSK acts on neural stem/progenitor cells (NSCs) during LV development is unknown. We demonstrated that TSK is crucial for the splicing and development-associated gene regulation of GFAP-expressing subventricular zone (SVZ) NSCs. We isolated GFAP-expressing NSCs from the SVZ of wild-type (GFAP^GFP/+^/TSK^+/+^) and TSK knock-out (GFAP^GFP/+^/TSK^−/−^) mice on postnatal day 3 and compared their transcriptome and splicing profiles. TSK deficiency in NSCs resulted in genome-wide missplicing (alteration in exon usage) and transcriptional dysregulation affecting the post-transcriptional regulatory processes (including splicing, cell cycle, and circadian rhythm) and developmental signaling networks specific to the cell (including Wnt, Sonic Hedgehog, and mTOR signaling). Furthermore, TSK deficiency prominently affected the splicing of genes encoding RNA and DNA binding proteins in the nervous SVZ and non-nervous muscle tissues. These results suggested that TSK is involved in the maintenance of correct splicing and gene regulation in GFAP-expressing NSCs, thereby protecting cell fate and LV development. Hence, our study provides a critical insight on hydrocephalus development.

## Introduction

Tsukushi (TSK) is a class IV small leucine-rich proteoglycan essential for development and metabolism (Ohta et al., 2004; Ahmad et al., 2018; Wang et al., 2019; Istiaq and Ohta, 2022). TSK has been linked to neurogenesis, neural circuit, eye development, inner ear development, bone growth, and liver homeostasis (Ohta et al., 2011; Yano et al., 2017; Wang et al., 2019; Ahmad et al., 2020; Miwa et al., 2020; Ito et al., 2021).

Lack of TSK disrupts brain development by affecting the morphology of the anterior commissure, dentate gyrus, corpus callosum, and lateral ventricle (LV) (Ito et al., 2010; Hossain et al., 2013; Ahmad et al., 2020 *).* Previously, we reported that TSK dysfunction was associated with hydrocephalus in mice and humans (Ito et al., 2021). Hydrocephalus is a life-threatening condition characterized by LV enlargement and high intracranial pressure. Moreover, TSK dysfunction resulted in LV enlargement and alterations in the neurogenesis of subventricular zone (SVZ) neural stem cells. During the crucial period of LV development, TSK deficiency leads to an increase in the number of neural stem/progenitor cells (NSCs) in newborns, which undergo excessive apoptosis on postnatal (P) day 10. Nonetheless, the molecular mechanism by which TSK influences NSCs’ fate remains unknown.

LV ependymal cells produce TSK during the initial stages of postnatal brain development. In the LV, TSK functions as an extracellular molecule that modulates Wnt signaling by binding to the Wnt receptor Frizzled3, hence influencing LV growth. Homologs of human and mouse TSK show an 85% similarity. Some patients with hydrocephalus have an exonic mutation in the *TSKU* gene. This pathological mutation disrupts structural integrity, rendering TSK unable to bind the Frizzled3 receptor and control Wnt signaling. Injecting a functional TSK protein into the murine LV at P0 can restore Wnt signaling and limit LV enlargement, hence reversing the hydrocephalic morphology. The LV cilia of TSK KO mice display an uneven, scattered distribution pattern, although this does not affect the cerebrospinal fluid flow. In addition, the TSK KO murine SVZ exhibits altered neurogenesis. In the P0 stage, the number of NSCs that express SOX2 increases, whereas in the P10 stage, the number of NSCs cells that express NESTIN and SOX2 increases but total SOX2 population decreases, along with the number of apoptotic cells in the SVZ. The number of progenitor cells that differentiate from neural stem cells expressing NESTIN in the later stages of development is similarly increased. TSK is also expressed in the hippocampus during early postnatal development (P0-P15). TSK deficiency affects the proliferation and differentiation of hippocampal neural stem cells by increasing the number of stem/progenitor cells and differentiated neuronal lineages, while decreasing the number of nonneuronal oligodendrocytes and astrocyte cells (Ahmad et al., 2020). Thus, TSK deficiency is characterized by the abnormal proliferation and differentiation of NSCs.

Of note, TSK is a multisignaling regulator. In addition to Wnt, TSK interferes with CCN2 and TGF-β1 signaling by interacting directly with CCN2 and TGF-β (Aoyama et al., 2012; Niimori et al., 2012; Hossain et al., 2013; Ohta et al., 2019). Furthermore, TSK binds to Frizzled4 and competes with WNT2b during peripheral eye development. TSK deficiency results in Wnt disruption in the peripheral eye, which causes ciliary body enlargement (Ohta et al., 2011).

TSK modulates effects on TGF-β signaling dependent on tissue context. In the control of the hair cycle, TSK binding to TGF-β1 promotes TGF-β1 signaling by increasing the expression of TGF-β1 and decreasing that of phosphorylated SMAD2/3. TSK deficiency causes delays in the hair cycle (Niimori et al., 2012). In contrast, TSK binding negatively inhibits TGF-β1 signaling during the wound healing process, thereby maintaining an immune response and inflammation (Niimori et al., 2014). TSK influences the netrin signaling pathway, ensuring that the axons of the anterior and posterior-anterior commissure cross the midline (Ito et al., 2010). Consequently, TSK is a suitable candidate for elucidating its probable connection with other signaling regulatory processes because of its participation in various signaling and regulatory cellular processes.

Hydrocephalus can be caused by the alteration of neural stem cell proliferation and differentiation in the SVZ niche (Carter et al., 2012; Li et al., 2014; Ohata and Alvarez-Buylla, 2016). During early postnatal development, GFAP is expressed by NSCs in the SVZ niche flanking the LV (Imura et al., 2003; Liu et al., 2006; Guo et al., 2013). In this study, we sought to determine the regulatory function of TSK in glial fibrillary acidic protein (GFAP)-expressing (GFAP^+^) NSCs in the early post-natal brain to understand the altered cell fate in the LV of hydrocephalic mice. We discovered that TSK modulated several development and fate determining signaling processes, such as Wnt, cell cycle, mTOR, SHH, and circadian rhythm. Unexpectedly, we also found that TSK is involved with posttranscriptional control by influencing the splicing process modulating the expression and splicing of spliceosome factors.

## Materials and methods

### Animals

TSK knock-out mice (TSK KO, TSK^−/−^) were generated as previously described (Ito et al., 2010). GFAP-EGFP (GFAP^GFP/+^, WT) knock-in mice were developed and provided by Dr. Shioda group at Center for Animal Resources and Development, Kumamoto University (Suzuki et al., 2003b; 2003a). TSK KO mice were mated with GFAP-EGFP knock-in mice to generate GFAP^GFP/+^/TSK^−/−^ (KO) mice. Desired offspring were selected for further expansion of the mouse colony. Mouse development stage P3 was used for the collection of SVZ flanking LV slices.

### Immunohistochemistry

Murine brains were collected from stage P3- Wt (TSK^+/+^) and TSK KO mice. Collected fresh brains were either immediately snap frozen in liquid nitrogen or fixed by 4% PFA at 4°C overnight. The fresh frozen brains were then embedded in OCT compound (Sakura Finetek) and frozen at −80°C. The fixed brain was submerged in 20% sucrose at 4°C for 24 h and then embedded in the OCT compound. Coronal sections (thickness: 30 μ m) were prepared by Cryostat (Leica Biosystem) machine and collected on glass slides. Collected section samples were incubated in blocking solution (5% skim milk or 5% heat inactivated normal goat serum and 0.1% Triton x-100 in PBS) for 1 h at room temperature (RT). Blocked sections were then incubated with primary antibodies for at least 16 h at 4°C and secondary antibody for 2.5 h at RT consecutively. Sections were washed by washing buffer (0.3% Triton x-100 in PBS) when necessary. The following primary antibodies were used: Monoclonal anti-TSK Mab1D3F1 (final concentration: 1 ug/ml, host: rat; previously validated originally developed antibody) (Ito et al., 2021), anti-GFAP (1:1000, host: chicken; Abcam), anti-GFAP (1:200, host: rat; ThermoFisher Scientific), anti-SOX2 (1:200, host: rabbit; Abcam), anti-S100β (1:50, host: rabbit; Abcam), anti-phospho (S235) -RPS6 (1:200, host: Rabbit; Abcam) antibody. The following Alexa flour secondary antibodies were used: anti-rabbit 647 (1:800; Abcam), anti-rat cy3 (1:1000; Jackson ImmunoResearch), anti-rat 488 (1:1000; Jackson ImmunoResearch) and anti-chicken 488 (1:1000; Jackson ImmunoResearch). Confocal images were obtained by Leica TCS SP8 STED (Leica Biosystem) confocal super-resolution microscope. Images were postprocessed by Leica LAS X software. All images were taken with a minimum pixel saturation. Staining and imaging conditions were similar during the comparison study. Each study included a control staining to check for non-specific signals from the secondary antibody (Supplementary Figure S1A, B). To count the GFAP/SOX2/S100β-expressing cells, two randomly selected SVZ coronal sections (4 SVZs) were chosen for each mouse. Positive cells were counted on the dorsal and medial sides of the SVZ spanning 80 μm width (from the LV wall) and 200 μm length (from the dorsal-medial junction in both direction). For the localization of TSK in GFAP^+^ cells, high-resolution Z-stack images (Z step size 0.33 μm) were acquired and analyzed by the 3D analysis tab in the LasX software. The intensity values for pS6 were calculated by subtracting the background signal from the obtained values. At least three or more mice were used for all the comparative studies. Wilcoxon rank sum test was used to estimate statistical significance.

### Cell sorting and flow cytometry

SVZ slices were dissociated into single cells using trypsin and pipetting. GFP expressing cells were sorted by fluorescence-activated flow cytometry (FACS). FACS Aria III (BD Biosciences) was used for cell sorting and flow cytometric analysis as previously described (Umemoto et al., 2006).

### RNA-sequencing

We used 100 sorted KO (nKO = 4) GFAP^GFP^ and WT (nWT = 4) GFAP^GFP^ murine cells and performed RNA-seq analysis using a modified version of the previously described approach (Hayashi et al., 2018). The cells were sorted directly into the lysis buffer, reverse transcription reaction and second strand synthesis were performed sequentially without RNA extraction minimizing the RNA loss. The PrimeScript RT reagent kit (Takara Bio Inc.) was used to synthesize the first strand of cDNA with “not-so-random” primers. For second-strand synthesis, Klenow fragment (3′–5′ exo-; New England Biolabs Inc.) and complement chains of “not-so-random” primers were used. To construct the RNA-seq library, pure double-stranded cDNA was synthesized and amplified using the Nextera XT DNA sample prep kit (Illumina Inc.). The Next-seq 500 machine (Illumina Inc.) was used to sequence the resulting library according to the manufacturer’s instructions. Every sample produced high-quality sequences. Sequence quality (Q score) was generally higher than 28 for the majority of raw reads. The low-quality sequences (Q < 20) were trimmed before downstream processing. On the sequence quality distribution curve, there was a peak at a Q score of 35 for all processed reads (Supplementary Figure S2).

### Transcript estimation and alternate splicing analysis

The acquired reads for each sample were mapped to the reference genome “GRCm39” provided by the ENSEMBLE database using the standard mood of the HISAT2 alignment program (Kim et al., 2019, 2). The level of transcript expression was estimated using the previously described HISAT, StringTie, and Ballgown methods, while StringTie 2.2.0 was run on reference guiding mood (Pertea et al., 2016). Downstream statistical analysis and visualization were performed according to the instructions by the Griffith lab (Supplementary Figure S3) (Griffith et al., 2015). For alternate splicing analysis, we considered the variation in exon usage between the test and control groups. Following a previously published technique, we used the DEXSeq 1.43.0 tool to statistically estimate the differential exon usage (DEU) in our data (False discovery rate, FDR <0.1) (Anders et al., 2012, 2013). For DEU estimation in bulk-RAN seq from LV and muscle tissue, we used FDR cutoff at 0.05.

### Enrichment, network, and protein-protein interaction analysis

The “ShinyGO v.0.75” program was used to perform gene set enrichment analysis using multiple databases available (FDR <0.05). Pathway analysis and visualization were performed using Pathview (Luo and Brouwer, 2013). Pathway enrichment analysis was performed using NetworkAnalyst (*p* < 0.05) (Xia et al., 2013, 2014, 2015; Zhou et al., 2019). String database-based protein-protein interaction network analysis was performed using string database, NetworkAnalyst and Cytoscape v. 3.8.1 (Shannon et al., 2003; Jensen et al., 2009; Zhou et al., 2019). Protein-protein docking between TSK and BUD31 was simulated using Patchdoc (Minimum clustering RMSD: 4) tool and PyMol version 2.5 software (Schneidman-Duhovny et al., 2005; Schrödinger, LLC, 2015a; 2015b). Protein structures were obtained from the AlphaFold database (Jumper et al., 2021).

## Results

### Tsukushi maintained GFAP^+^ NSCs pool in the post-natal subventricular zone

**FIGURE 1:**
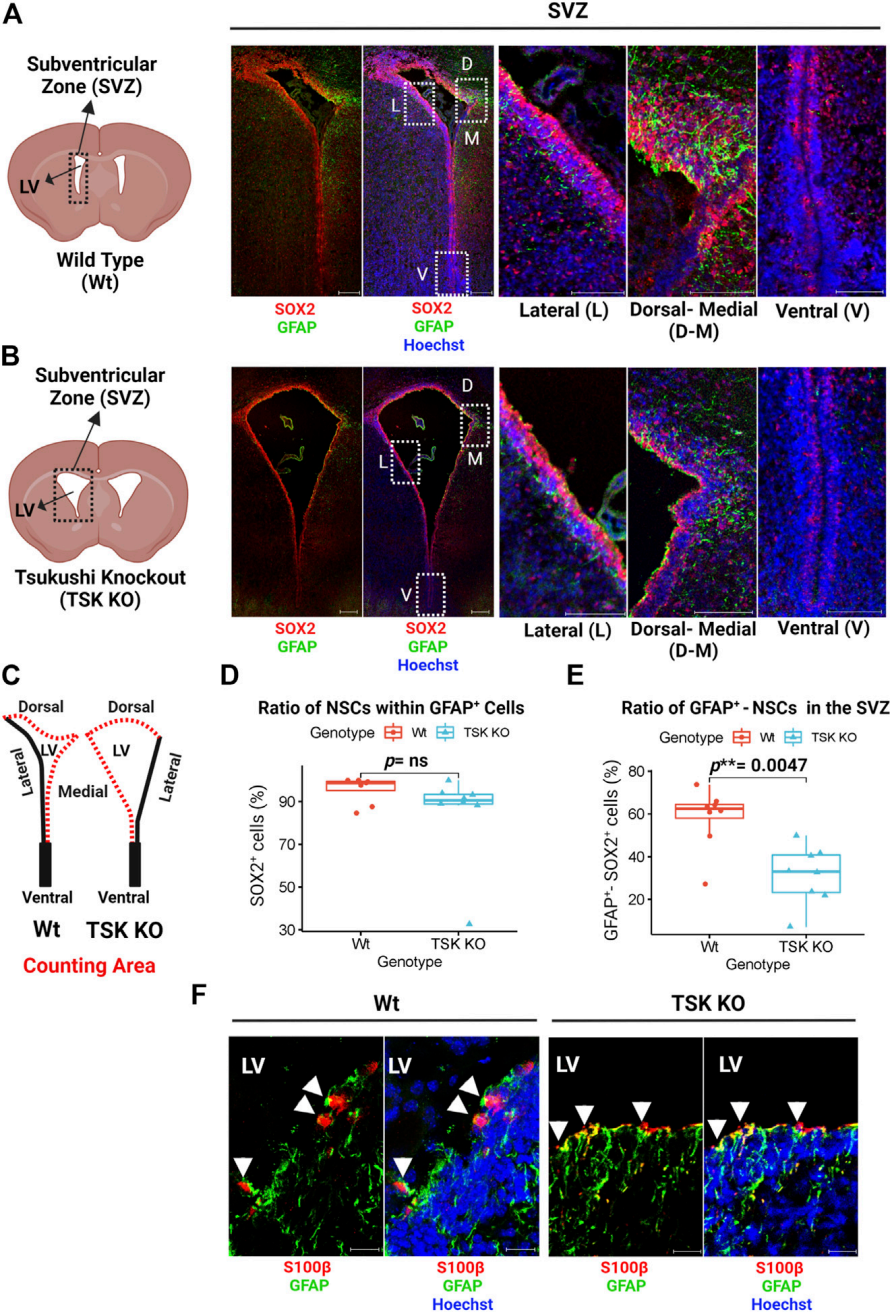
Tsukushi deficiency disrupts GFAP+ NSCs. **(A)** Representing cartoon and images of P3 stage Wt SVZ immunostained for GFAP (Green), SOX2 (Red) and Nuclei (Hoechst, Blue). Scale bar = 200 μm (whole SVZ),100 μm (enlarged views). **(B)** Representing cartoon and images of P3 stage TSK KO SVZ immunostained for GFAP (Green), SOX2 (Red) and Nuclei (Hoechst, blue). Scale bar = 200 μm (whole SVZ),100 μm (enlarged views). **(C)** Cartoons of Wt and TSK KO SVZ showing the area for cell counting. **(D)** Percentage distribution of SOX2^+^ cells within GFAP^+^ cell population in SVZ. Distribution is shown by box plot with jitters. Each dot represents a SVZ. Number of brain samples: nTSK KO = 4, nWt = 4. **(E)** Percentage distribution for the GFAP^+^-SOX2^+^ double positive SVZ cells. Distribution is shown by box plot with jitters. Each dot represents a SVZ. Number of brain samples: nTSK KO = 4, nWT = 4. **(F)** IHC analysis for GFAP (Green) expression in S100β^+^ (Red) cells in SVZ at P3. Cell nuclei are stained with Hoechst (Blue). Arrowhead indicates GFAP^+^-S100β^+^ double positive cells. Scale bar 20 μm. LV = Lateral ventricle. The image focuses on the medial side of the SVZ. All the statistical significance is denoted by *p*-value using Wilcoxon rank sum test. P *< 0.05, p**< 0.01, p***< 0.001, *p* ≥ 0.05 (ns = not significant).

NSC proliferation and apoptosis are affected by TSK deficiency during postnatal neurogenesis (Ahmad et al., 2020; Ito et al., 2021; Istiaq and Ohta, 2022). It is unknown, however, how this affects the cellular homeostasis and functional potential of SVZ NSCs. During early postnatal brain development, NSCs exhibit astrocyte-like characteristics, expressing GFAP, which later develop into mature astrocytes (Alves et al., 2002; Merkle et al., 2004; Matsubara et al., 2021). In order to determine whether TSK dysfunction affects these GFAP^+^ NSCs in the early postnatal murine brain, we stained the SVZ (P3) with GFAP and SOX2 (NSC marker) by immunohistochemistry. We found that GFAP is abundantly expressed in the dorsal and medial sides, while the lateral and ventral sides exhibit very little or no expression in Wt and Tsukushi knockout mice (Figures 1A,B). In SVZ cells, GFAP co-expressed with SOX2. We examined the proportion of SOX2 expressing (SOX2^+^) cells within the GFAP^+^ cell population in the SVZ. No significant differences were found between Wt and TSK KO mice (Figures 1C,D). Next, the percentage of SVZ cells (dorsal and medial) that were positive for both GFAP and SOX2 markers (GFAP^+^-SOX2^+^), were compared between Wt and TSK KO. TSK KO mice SVZ showed a significant decrease in GFAP^+^-SOX2^+^ cells (p** = 0.0047) (Figure 1E). In addition to NSCs, the SVZ also contains ependymal cells that express SOX2 (Shah et al., 2018). In the early stages of ependyma development, GFAP is expressed by ependymal progenitor cells (MacDonald et al., 2021). Though mature ependymal cells do not express GFAP, no definitive research has been conducted on GFAP expression on ependymal cells at P3. Accordingly, we examined GFAP expression in ependymal cells using the S100β as an ependymal marker. Both Wt and TSK mice showed a subpopulation of S100β expressing (S100β^+^) cells that expressed GFAP (Figure 1F). These data support that TSK deficiency affected the fate of GFAP^+^ NSCs which may generate GFAP^+^-S100β^+^ependymal cells.

**FIGURE 2:**
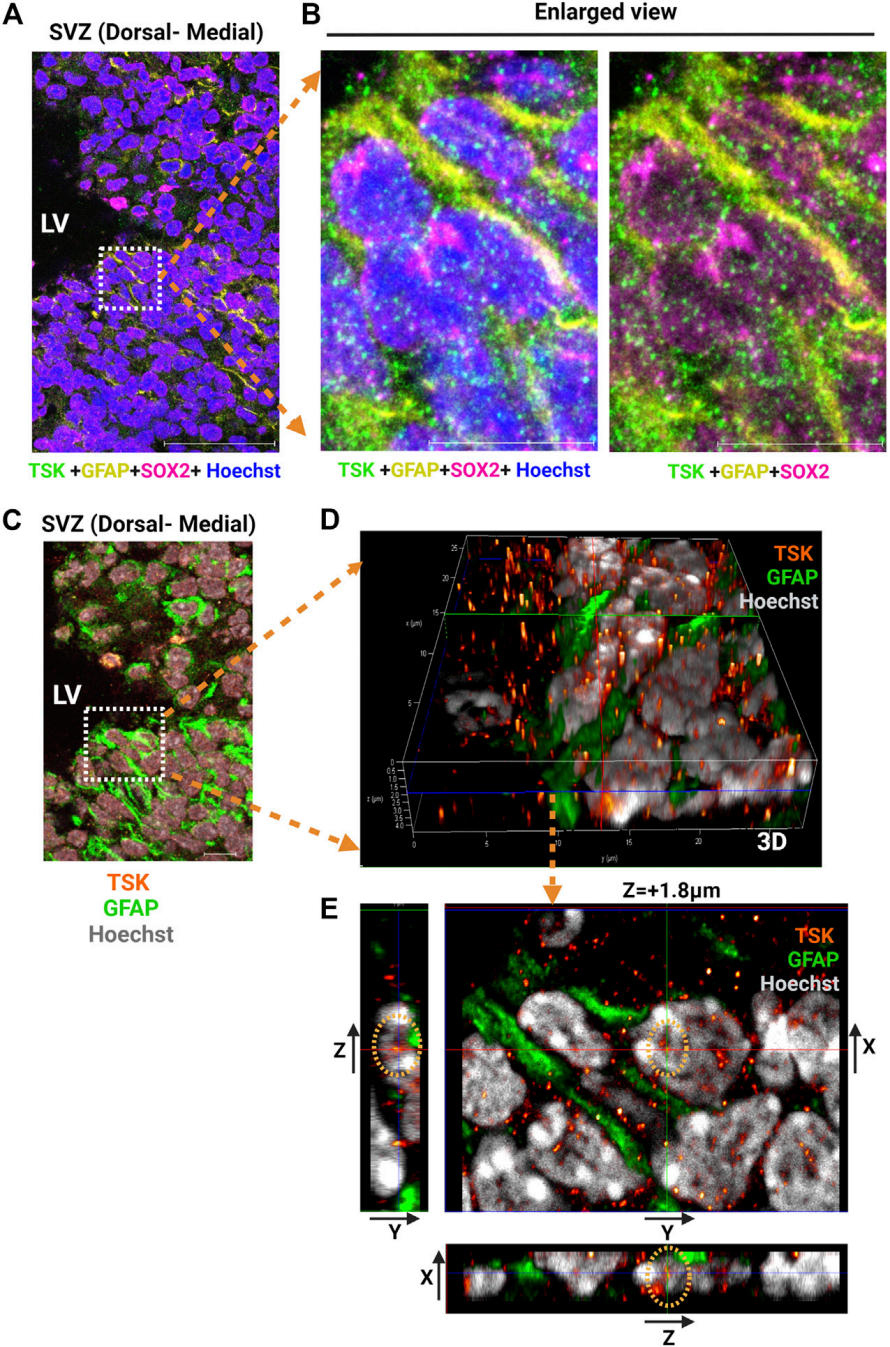
GFAP+ NSCs exhibit nuclear localization of TSK. **(A)** Representative image of SVZ from P3 stage Wt immunostained for TSK (Green), GFAP (Yellow), SOX2 (Magenta) and Nuclei (Hoechst, Blue). Scale bar = 50 μm. **(B)** Enlarged views of the highlighted area in **(A)** showing the localization of TSK within the various cellular compartments. Scale bar = 10 μm. **(C)** Representative Z-stack merged image of SVZ from P3 stage Wt immunostained for TSK (Orange gradient, glow), GFAP (Green), and Nuclei (Hoechst, Grey). Image obtained from the same SVZ section as **(A)**. Z step size = 0.33 μm. Scale bar = 50 μm. **(D)** Enlarged views of the highlighted area in C showing the three-dimensional (3D) localization of TSK in the nuclear compartments of GFAP^+^ cells. Colored lines indicate focus point from different axis slices (Red = Y-axis, Green = X-axis and Blue = Z-axis). **(E)** Snapshot of the analysis showing TSK localization in the GFAP^+^ cell nucleus. The image shows the focus plane at a depth of approximately 1.8 μm from the surface of the nucleus. Images of the TSK signal from the Y-X axis, the Z-Y axis, and the X-Z axis are displayed in the split images.

Previously, we reported that TSK is secreted by ependymal cells, binds to frizzled three receptors, and regulates Wnt signaling. We hypothesized that the secreted TSK may affect Wnt signaling non-autonomously in the SVZ cells (Ito et al., 2021; Quaresima et al., 2022). To investigate how TSK impacts GFAP^+^ cells, we analyzed the localization of TSK in GFAP^+^ cells by immunohistochemistry using monoclonal anti-TSK antibody (Mab1D3F1)**.** In GFAP^+^-SOX2^+^ cells, TSK was found inside the nucleus in addition to the extracellular space (Figures 2A–E; Supplementary Figure S1B; Supplementary Video S1). In order to confirm that TSK expression is present in non-ependymal cells, we looked at the adult murine SVZ single cell RNA-seq data (Zywitza et al., 2018). The TSK gene was expressed by various non-ependymal cells within the adult SVZ, including astrocytes, neuroblasts, and oligodendrocyte progenitor cells (Supplementary Figure S4). Based on these results, TSK may also play an intranuclear role in the GFAP^+^ cells in addition to providing extracellular signaling activity.

### Tsukushi deficiency influenced key developmental and posttranscriptional regulatory pathways in GFAP^+^ cells

**FIGURE 3:**
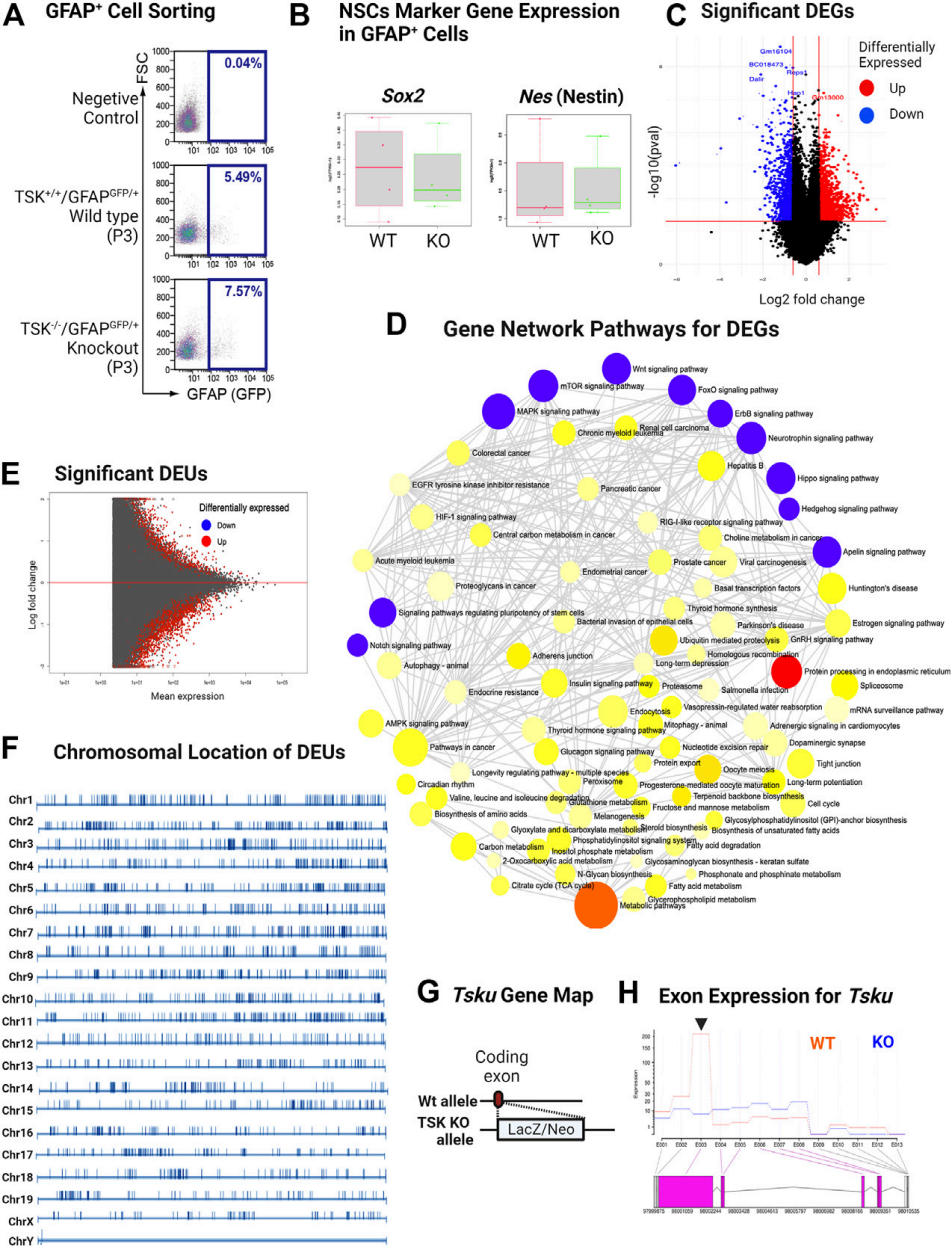
KO GFAP+ cells exhibit differential gene expression in multiple developmental pathways and genome-wide missplicing of coding genes. **(A)** Representative image of sorting of GFAP^GFP+^ cells by FACS. **(B)** FPKM distribution of *Sox2* and *Nes* NSC markers in WT and KO groups. **(C)** Volcano plot of differentially expressed genes. Genes with significant (*p* < 0.05) increase and decrease in their expression are colored in red and blue, respectively. **(D)** Gene-set enrichment network of differentially expressed genes using significantly enriched KEGG pathway functional categories (*p* < 0.05). The nodes represent enriched pathway gene sets. The size of the node is proportional to the number of relevant genes in the set. The *p*-value of the node is indicated by the yellow to red gradient. Essential developmental signaling pathways are indicated by blue colored nodes (irrespective of *p*-value). Edges (gray lines) indicate shared genes between two nodes. **(E)** MA plot for significant differential exon usage between the wild-type and knock-out groups. Significant hits at FDR = 0.1 are colored in red. **(F)** Chromosomal distribution of significant DEU-coding genes. The position of DEU genes is marked by vertical bars. The scale differs for each chromosome. **(G)** Schematic of TSK KO generation by allelic insertion of LacZ/Neo cassette in the coding exon. **(H)** Expression of *Tsku* exons in WT vs. KO. Data shown as Fitted expression plot. Pink indicates the significant differentially expressed coding exon. Arrowhead indicates the coding exon of *Tsku* (Targets for LacZ/Neo cassette insertion). The data presented here was generated by comparing four biological replicates from the WT and KO groups.

To understand the regulatory role TSK in the GFAP^+^ cells, we crossed TSK KO mice with WT (GFAP^GFP/+^) mice and generated KO (GFAP^GFP/+^/TSK^−/−^) mice. We sorted GFAP^GFP^ cells from the LV lining slices of KO (nKO = 4) and WT (nWT = 4) mice at P3 (Figure 3A). We did not find any significant differences in the number of GFAP^+^ cells between KO and WT groups in the sorting data. However, we noticed that cell counts in the KO group were more clustered around the mean (standard deviation = 1.45) compared with the WT group (standard deviation = 4.5), suggesting that TSK deficiency had a robust impact on the number of GFAP^+^ cells in the LV as we observed in TSK KO mice (Supplementary Table S1). To investigate the involvement of TSK in transcriptional regulation, 100 sorted cells from each mouse were analyzed using an ultralow input RNA-sequencing technique. The technique can be used for determining gene expression of cells that exist in low numbers and for in-depth transcript analysis, such as splicing (Hayashi et al., 2018). We employed a comprehensive RNA-seq analytical method that incorporates splice variation during the estimation of the transcriptome, using HISAT, StringTie, and Ballgown tools (Pertea et al., 2016). Both WT and KO GFAP^+^ cells expressed mRNA for NSC markers SOX2 (*Sox2*) and NESTIN (*Nes*), indicating that the sorted cells represent the SVZ NSC population (Figure 3B). Transcriptome estimation by StringTie and relevant gene inference by Ballgown showed that 2666 genes were differentially expressed (differentially expressed genes, DEGs) between the KO and WT groups to a significant (*p* < 0.05) degree (Figure 3C). Among DEGs, we detected that 62% (1648) had increased expression, whereas 28% (1018) had decreased expression (Supplementary Material S1, 2). To understand which pathway these DEGs belonged to, we performed gene set pathway enrichment network analysis. The significant enriched nodes of the network showed a number of interrelated pathways involved in cell regulation, stemness, and development. Notably, we detected Wnt, hedgehog, mTOR, hippo, FOXO, apelin, and other key developmental signaling pathways (Figure 3D; Supplementary Table S2). These pathways are known for their roles in morphological development, ciliogenesis, NSC maintenance, and cell fate determination (Polter et al., 2009; Lipton and Sahin, 2014; Cheng et al., 2020; Lv et al., 2020; Da Silva et al., 2021). In addition, we also identified neurodegenerative disease- and cancer-associated pathways in the network, suggesting a possible transcriptional similarity between TSK deficient GFAP^+^ cells and cells of these diseases. In the course of transcriptome mapping and DEG estimation, we observed that a number of novel transcripts were included in the DEG list in addition to known transcripts for the genes (Supplementary Material S1). Hence, the KO group may have exhibited splicing errors. Therefore, we posited that TSK might also influence RNA splicing. Alternate splicing can occur in multiple forms, including splicing site variation, intron retention, and exon modification (WANG et al., 2015). In our study, we opted for an exon-based strategy because it identifies isoform variants. Transcript isoform variation is more likely to influence protein structure or function, thereby influencing cellular processes. We applied a statistical approach (DEXseq) to estimate the differential exon usage between WT and KO GFAP^+^ cells. DEXseq analysis revealed genome-wide missplicing due to differential exon usage in the KO group. Notably, we identified 3707 genes with differential exon usage (DEU) (Figure 3E). The chromosomal positions of the DEU coding genes indicated that they were distributed in all the chromosomes indicating a global splicing anomaly (Figure 3F; Supplementary Material S3). In order to confirm that such events are indeed caused by the knockdown of TSK in GFAP^+^ cells, we estimated the exonic expression of *Tsku* gene in the WT and KO groups. To generate TSK KO, LacZ/Neo cassette were inserted into the coding exon’s HindIII-restriction-enzyme-cutting site that creates a null mutant TSK allele (Ito et al., 2010). Our results showed that the WT GFAP^+^ cells encoded functional TSK, while the KO cells had null mutant alleles encoding beta-galactosidase (encoded by LacZ insertion) having almost no expression of functional coding exons (Figures 3G,H). Thus, the observed phenomenon was due to the absence of functional TSK in KO GFAP^+^ cells.

**FIGURE 4:**
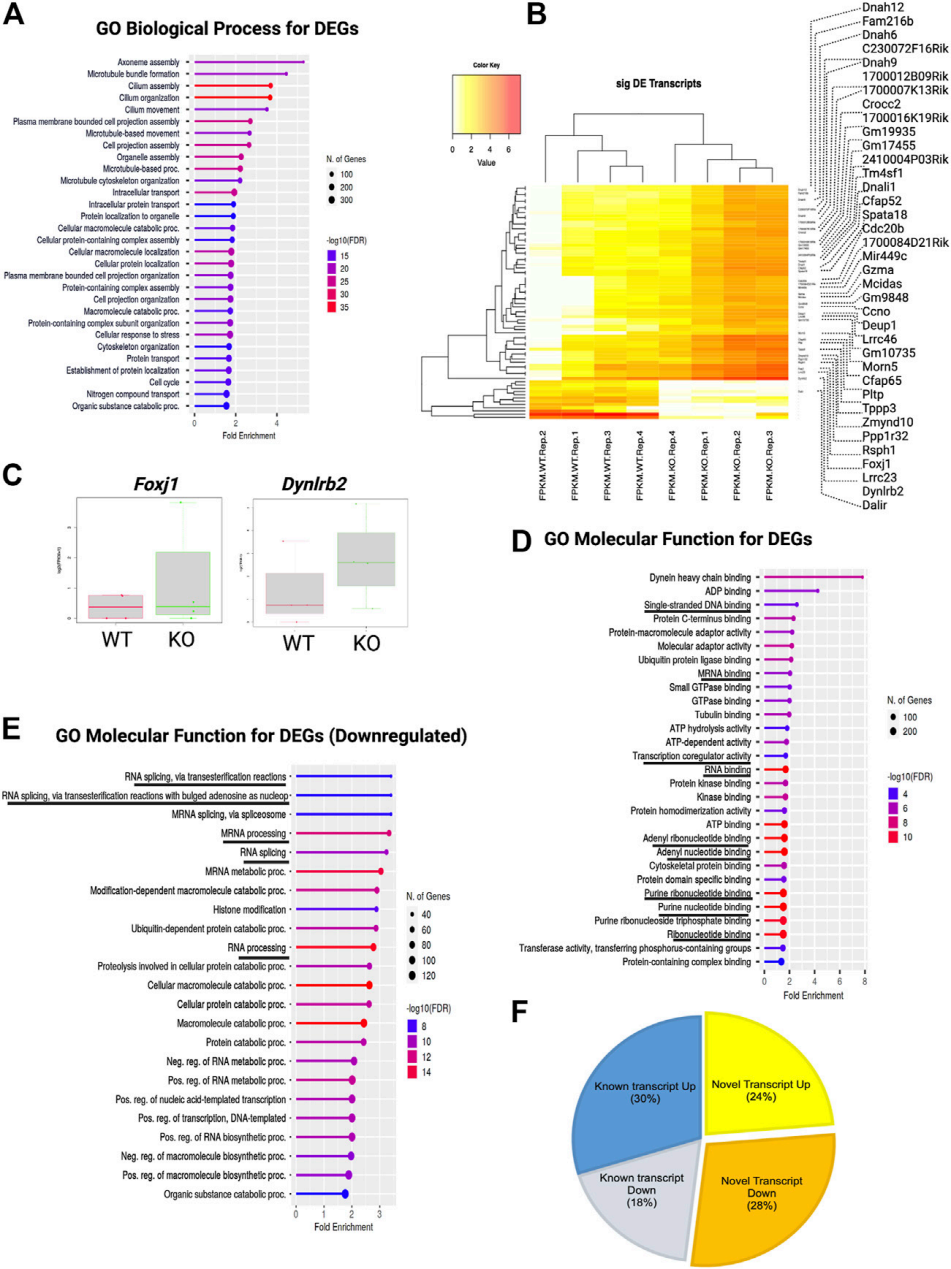
TSK deficiency dysregulate ependymal-like genes, posttranscriptional regulation, and levels of variant transcripts. **(A)** Top 30 GO biological process categories significantly enriched in DEGs. The blue to red gradient of the lollipop indicates-log FDR value. The length of the lollipop indicates fold enrichment. The size of the lollipop tip indicates the number of positive gene hits in relevant categories. **(B)** Heatmap of significant (*p* < 0.05) differentially expressed (DE) transcripts (Log2FC ≥ 2) between WT and KO samples. The yellow to red gradient indicates the level of expression. Unnamed rows in the heatmap gene column indicate novel transcripts. **(C)** FPKM distribution of *Foxj1* and *Dynlrb1* ependymal cell markers in WT and KO groups. **(D)** Top 30 GO molecular function categories significantly enriched in DEGs. A black underline indicates terms associated with nucleic acid binding. **(E)** Top 30 GO molecular function categories significantly enriched in DEGs with decreased expression. A black underline indicates terms associated with posttranscriptional regulation-related RNA binding. **(F)** Percentage distribution of novel and known transcripts in differentially expressed transcripts. Up and down indicates increase and decrease of the expression respectively.

Next, we categorized these DEGs using gene ontology (GO) and performed overrepresentation-based enrichment analysis. We found that these DEGs were enriched in mostly ependymal cell-related GO biological process terms, such as axoneme assembly, microtubule bundle formation, cilium assembly, intracellular transport, macromolecule metabolic processes, and cell cycle (Figure 4A) (False discovery rate, FDR <0.05). Therefore, we checked the ependymal cell-related markers and found that the expression of both FOXJ1 transcription factor and DYNLRB2 was increased in KO GFAP^+^ cells, supporting the interpretation that a possible dysregulation of the transcription process was occurring in the ependymal GFAP^+^ subpopulation (Figures 4B,C). However, we didn’t find any significant difference in the critical NSCs markers such as *Gfap, Sox2 or Nestin* (Supplementary Material S1). Prominently, from the GO molecular function enrichment analysis, we detected nucleic acid-binding terms, suggesting transcriptional regulatory genes among DEGs (Figure 4D). We further performed enrichment analysis separately for the DEGs with increased and decreased expression, respectively. Surprisingly, we found that DEGs with decreased expression were associated with posttranscriptional regulation-related GO terms, such as mRNA processing, RNA splicing, and mRNA metabolic process, indicating a loss of transcriptional regulation in KO GFAP^+^ cells (Figure 4E).

These results suggested that the decreased expression of transcriptional regulators in KO GFAP^+^ cells might result in the upregulation of DEGs involved in critical neural stem cell pathways and ciliogenesis, thereby altering the proliferative and differentiation capacities of these cells. This suggested that TSK plays a role in transcriptional and post-transcriptional regulation.

### Tsukushi was required for proper splicing

**FIGURE 5:**
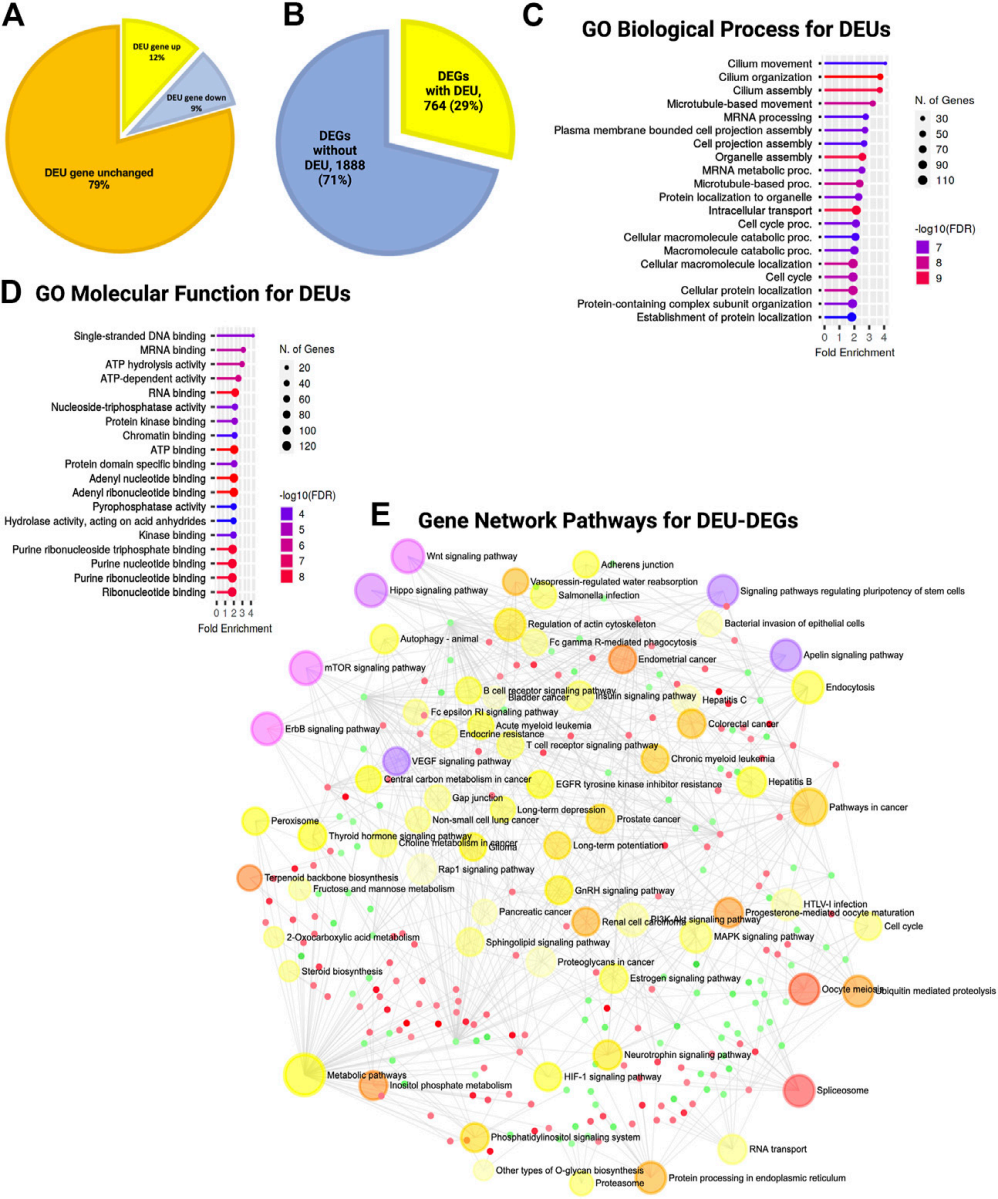
TSK deficiency disrupts the splicing of genes associated with ciliary function, transcriptional regulation, and developmental pathways. **(A)** Percentage distribution of DEU genes with differential gene expression. The color of the pi-chart slice corresponds to its annotation. **(B)** Percentage distribution of DEGs with DEU. **(C)** Top 20 GO biological process categories significantly enriched in DEGs with DEU. The blue to red gradient of the lollipop indicates the-log FDR value. The length of the lollipop indicates fold enrichment. The size of the lollipop tip indicates the number of positive gene hits in relevant categories. **(D)** Top 20 GO molecular function categories significantly enriched in DEGs with DEU. **(E)** Gene set enrichment network of differentially expressed genes that have DEU using significantly enriched KEGG pathway functional categories (*p* < 0.05). The larger nodes represent enriched pathway gene sets. The smaller nodes correspond to individual genes. The size of the node is proportional to the number of relevant genes in the set. The *p*-value of the node is indicated by the yellow to red gradient. Essential developmental signaling pathways are indicated by the violate gradient, with darker shades indicating higher *p* values. Edges (gray lines) indicate shared genes between two nodes. The network is represented as a bipartite network.

During the estimation of transcripts, we detected that a large portion (52%) of the differentially expressed transcripts (DETs) were novel and could not be assigned to any genes in the reference genome (GRC m39). Of the total DETs, 24% of those with increased expression and 28% of those with decreased expression were novel (Figure 4F; Supplementary Material S1). We assigned and analyzed the remaining 48% of transcripts to known genes. This substantial percentage of novel or unknown DETs in KO GFAP^+^ cells clearly indicated a splicing error. Among all DEU genes, we detected that 12% had increased expression, 9% had decreased expression, and 79% showed no significant change in the level of gene expression between the WT and KO groups (Figure 5A). Cross-analysis of the DEGs with the DEU gene data revealed that 29% of the DEGs with increased expression and 71% of those with decreased expression were DEU genes (Figure 5B). GO term enrichment analysis of DEU genes revealed that DEU genes were abundant in posttranscriptional regulatory mechanisms, including mRNA processing, RNA splicing, and chromosomal organization (Figure 5C). These enriched DEU genes predominantly encode RNA- and DNA-binding proteins, indicating that alternative transcripts through varied exon use might modify their essential functions in the transcriptional and posttranscriptional control of gene expression (Figure 5D). Plotting these genes in the pathway enrichment network revealed their functional role in transcriptional regulation, metabolism, and key developmental signaling pathways (Wnt, hippo, mTOR, apelin, etc.). Of note, we detected the most significant enrichment in the spliceosome, which is known to be responsible for intron splicing and exon selection during pre-mRNA processing (Figure 5E; Supplementary Table S3). Thus, we assumed that TSK is necessary for the correct splicing of developmental signaling-associated genes and transcriptional regulation in GFAP^+^ NSCs.

**FIGURE 6:**
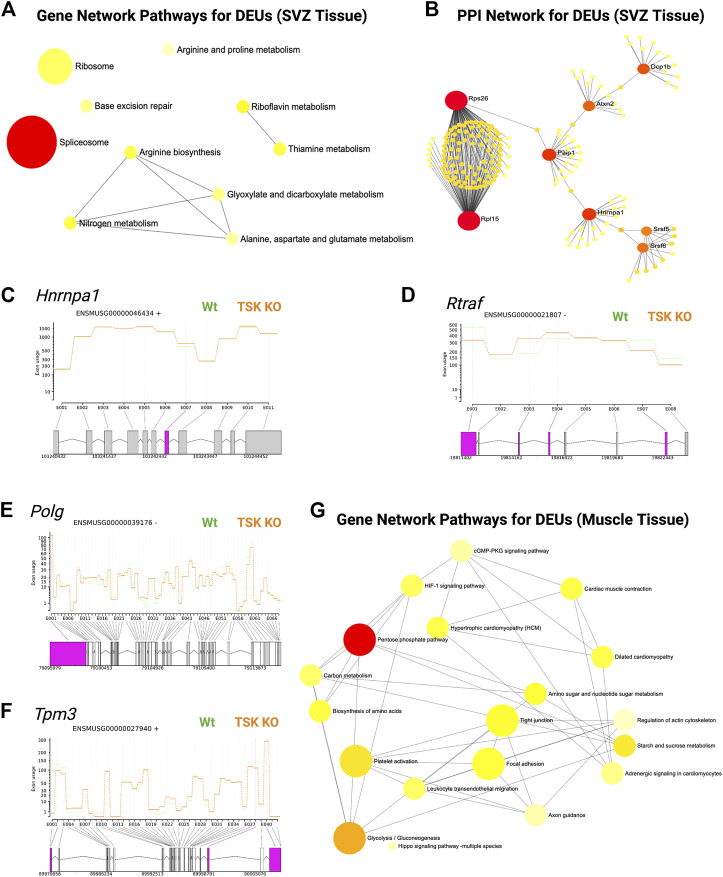
TSK regulates the splicing in SVZ and muscle tissues. **(A)** Gene-set enrichment network of SVZ genes with DEU using significantly enriched KEGG pathway functional categories (*p* < 0.05). The nodes represent enriched pathway gene sets. The size of the node is proportional to the number of relevant genes in the set. The *p*-value of the node is indicated by the yellow to red gradient. Edges (gray lines) indicate shared genes between two nodes. **(B)** Network topology of spliceosome SVZ DEU genes by string database-based analysis of protein-protein interactions (PPI). Interaction was estimated for a confidence score cutoff = 900. All interactions were based on the evidence of physical interactions. Each nodes indicate a protein. The size of the nodes is proportional to their degree values. The color (yellow to red gradient) of the nodes are based on their betweenness centrality value. Edges (gray line) indicates interaction between two nodes **(C)** Fitted splicing plot for representative spliceosome associated SVZ DEU gene. Pink indicates the significant differentially expressed exon. **(D)** Fitted splicing plot for posttranscriptional regulatory RNA binding DEU gene. Plot is annotated similar to that in (C). **(E)** Fitted splicing plot for representative transcription regulatory DNA binding DEU genes in muscle tissue. Plot is annotated similar to that in (C). **(F)** Fitted splicing plot for representative myofibrillar gene in muscle tissue. Plot is annotated similar to that in (C). **(G)** Gene-set enrichment network of genes with DEU in muscle tissue using significantly enriched KEGG pathway functional categories (*p* < 0.05). Plot is annotated similar to that in (A).

In addition, we performed DEU analysis of the previously published bulk RNA-seq data from murine LV slices (representing GFAP expressing and non-expressing diverse cell populations in the SVZ of TSK KO and Wt mice at P3 stage), which revealed similar significant gene enrichment (GO molecular functions) in the DNA binding, RNA binding, and splicing related categories (Figure 6A; Supplementary Material S4). Protein-protein interaction analysis showed that the hub genes were involved in the posttranscriptional regulation that can affect the splicing and translational process in SVZ niche (Figures 6B–D; Supplementary Table S4, 5). However, we did not observe any significant DEU for important development associated genes. These results suggest that TSK is also involved in the posttranscriptional regulation of splicing in GFAP non-expressing cells of the LV compartment, and the consequence of missplicing is likely to be cell type dependent.

Moreover, we also investigated whether TSK promotes correct splicing in tissues other than in the brain. We analyzed publicly available RNA-seq data from TSK-KO murine muscle tissue (Wang et al., 2022). DEU analysis showed significant missplicing at 24 coding genes that have functions in transcription (*Polg, Zfp708, Rslcan18*), signaling (*Gpi1, Insr*)*,* apoptosis regulation (*AEN*), and muscle contraction (*Tpm3, Myl6, Myl12b, Myl12b*) (Figures 6E,F; Supplementary Material S5). Network enrichment analysis suggested that these misspliced genes can influence the metabolism (pentose phosphate, glycolysis), signaling (HIF-1, cGMP-PKG) and myofibrillary functions (muscle contraction, regulation of actin cytoskeleton) (Figure 6G; Supplementary Table S6). Therefore, TSK may play a role in maintaining appropriate splicing and signaling processes in other tissues of the body as well.

### Tsukushi regulated the splicing and gene expression of spliceosome factors

**FIGURE 7:**
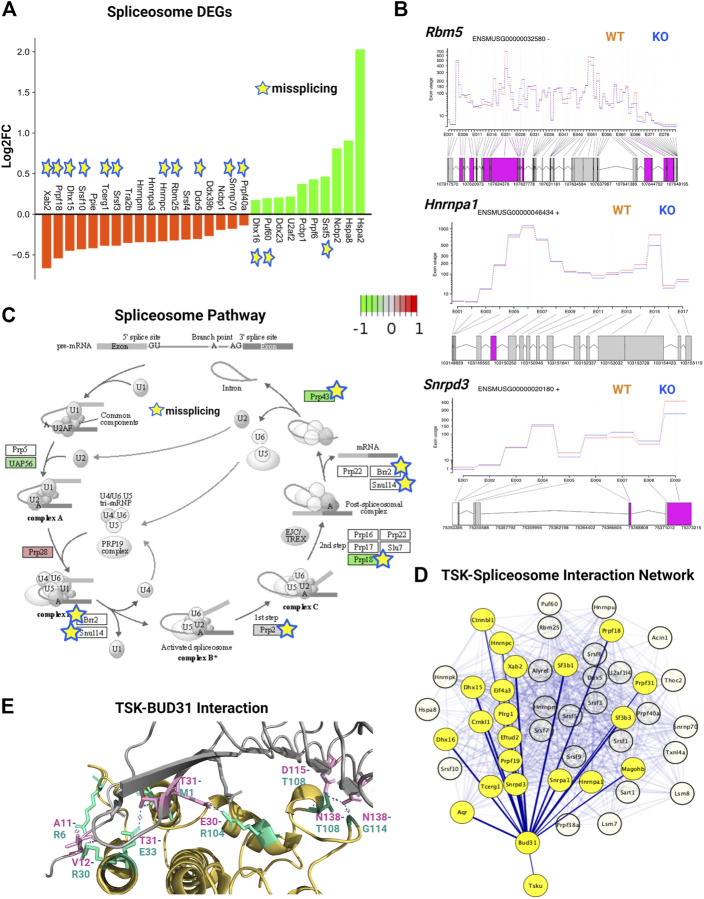
TSK deficiency alters spliceosome integrity. **(A)** Expression of spliceosome-associated DEGs. Green indicates increased expression, whereas orange indicates decreased expression. Star indicates misspliced proteins inferred from the DEU genes. The boxes without any color or star indicate that the pathway component does not correspond to DEG or DEU genes. **(B)** Fitted splicing plot of DEU in representative spliceosome-associated genes. Pink indicates the exon with significant differential exon usage. **(C)** Spliceosome pathway map for associated DEGs and DEU genes. Each rectangular box indicates a protein component of the pathway. Arrow indicates the activation targets of the pathway components. The green to red gradient indicates the level of expression. Star indicates the proteins inferred from the misspliced encoding genes. **(D)** String-based analysis of protein-protein interactions of TSK with DEU spliceosome genes. The nodes represent proteins. Yellow colored nodes represent the first neighbors of TSK-interacting proteins Bud31. The thickness of the edges is proportional to their confidence scores (Minimum confidence score for the interaction = 0.4). **(E)** Interaction surface between TSK and spliceosome factor BUD31. The contacting amino acids are shown in magenta (TSK) and cyan (Bud31) sticks. Interacting amino acid (with position) pair (TSK-BUD31) are shown in respective colors. Dotted lines between interacting atoms represent polar contacts. The number value in the polar contact lines indicates the distance between the interacting pair in Angstrom unit.

We analyzed the expression and DEU of spliceosome-associated genes. We found that half of the spliceosome-related DEGs (14 of 28) were misspliced, whereas the majority of genes had decreased expression (18 of 28) (Figure 7A). Next, we categorized DEU genes based on the number of observed exon changes (Supplementary Material S3). We found that the *Rbm5* spliceosome gene had the highest number of exon alterations (Figure 7B). We further identified that key spliceosome-associated genes, such as *Snrnp70*, *Prpf40a*, *Ddx5*, *Dhx50*, *Rbm25*, *Hnmpc* were misspliced and had decreased expression, whereas *Srsf5*, *Puf60*, and *Dhx16* were misspliced and had increased expression. We also detected other key spliceosome-associated non-DEGs (*Hnrnpa1*, *Srnpd3*, and *Brr2*) that were misspliced (Figure 7B; Supplementary Material S3). From the pathway analysis, we found that the decrease in the expression of the spliceosome component gene (U2) and overexpression of *Prp28* affected the proper formation of the spliceosome complex. For instance, we observed that missplicing and decreased expression of *Brr2* and *Smull4* affected spliceosome activation, whereas missplicing of *Prp2* and *Prp18* impaired spliceosome complex-RNA binding, and finally, the decreased expression of *Prp43* impacted the reactivation cycle of the process (Figure 7C). Hence, the overall missplicing and differential expression of these genes impacted every step of the spliceosome function, leading to a genome-missplicing signature.

To predict the mechanism by which TSK controls splicing, we performed a protein-protein interaction (PPI) network analysis of TSK with DEU spliceosome-associated genes. Our PPI analysis predicted that TSK might interact with BUD31 (confidence score = 0.41), which in turn interacts with key spliceosome component proteins such as SRNPD3, HNRMPA1, DHX15, and CTNBL1 to promote the correct splicing process. TSK has a putative homology (confidence score = 0.22) with LEA1, which binds to the BUD31 spliceosome protein, thus suggesting that TSK might interact with DUB31 to maintain the spliceosome process (Figure 7D; Supplementary Material S6). Therefore, we conducted a protein-protein docking simulation for TSK and BUD31. Based on the analysis, BUD31 is predicted to bind to TSK near the N-terminal region with eight polar interactions (Figure 7E). Besides altering exon selection, misssplicing may also occur by varying the 5′ or 3′ prime splice sites (WANG et al., 2015). However, TSK dysfunction did not significantly affect the 5′ or 3′ prime splice sites in SVZ GFAP expressing cells (estimated by MATS software 2.1.0). Hence, TSK may play an exclusive role in exon selection during splicing.

In the DEU analysis of SVZ tissue comprising diverse cell types, we also found that TSK deficiency altered the splicing of spliceosome factors (*Paip1*, *Hnrmpa1*, *Srsf5*, and *Srsf6*). In addition, analysis of the protein-protein interactions of the DEU genes indicated that missplicing could have altered the splicing (*Hnrnpa1, Srsf5, Srsf6*) and translational regulation (*Rps26, Rpl15*) (Figures 6A–D). Therefore, these data suggested that nuclear TSK plays a role in posttranscriptional regulation by safeguarding the splicing process in SVZ NSCs.

### Tsukushi regulated Wnt signaling and cell cycle

**FIGURE 8:**
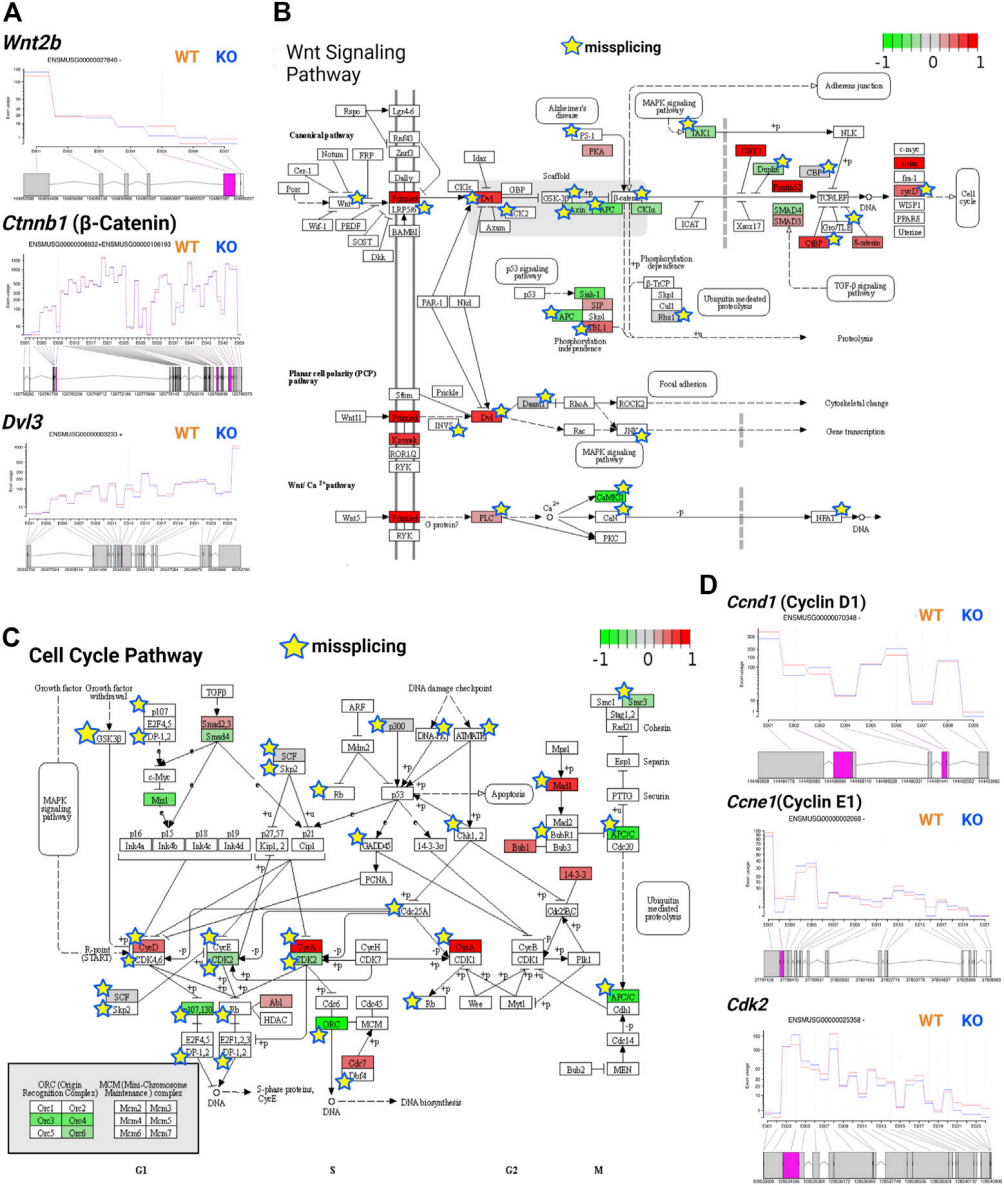
TSK deficiency impairs Wnt signaling and cell cycle. **(A)** Fitted splicing plot for DEU in representative Wnt pathway-associated genes. Pink indicates the exon with significant differential exon usage. **(B)** Wnt pathway map for associated DEGs and DEU genes. Each rectangular box indicates a protein component of the pathway. Arrow indicates the activation targets of the pathway components. Blunt arrow indicates the inhibition targets of the pathway components. The green to red gradient indicates the level of expression. Star indicates the misspliced proteins inferred from the associated DEU genes. The boxes without any color or star indicate that the pathway component does not correspond to DEG or DEU genes. **(C)** Cell cycle pathway map for DEGs and DEU genes. Annotated similar to the previous plot (B). **(D)** Fitted splicing plot for DEU in representative cell cycle pathway-associated genes. Plot is annotated similar to that in (A).

Wnt signaling is tightly regulated during development as it is involved in embryonic development, axis patterning, neural stem cell fate determination, and other key developmental processes (Gilbert, 2000). During development, Wnt signaling in the ventricular zone maintains the radial glial cell population and differentiation of intermediate progenitors in the subventricular zone (Chodelkova et al., 2018). Wnt signaling is triggered when Wnt proteins bind to the Frizzled receptor, activating *Dvl* genes and regulating transcription *via* canonical (β-catenin-dependent) or noncanonical (β-catenin-independent) pathways (Gilbert, 2000). Previously, TSK was found to inhibit Wnt signaling in the LV by binding to the Frizzled receptor (Ito et al., 2021). We observed that TSK deficiency in GFAP^+^ cells overactivated the Wnt signaling pathway by significantly increasing the expression of Frizzled, DVL, and cyclin D encoding genes, although Wnt and β-catenin encoding genes did not change significantly between WT and KO mice. We further found that the expression of Wnt regulators axin, APC, and SMAD4 was decreased, whereas that of CBY1, Pontin52, CTBP, and δ-catenin was increased, suggesting an impairment in the regulation of Wnt signaling (Supplementary Table S7; Supplementary Material S1). In addition, we detected that key Wnt signaling genes, such as *Wnt2b*, *Wnt7b*, *Ctnnb1*, *Dvl3*, axin, and *LRP5/6*, showed missplicing signatures in single or multiple exons (Figure 8A). Consequently, pathway analysis showed that both canonical and noncanonical Wnt pathways might be overactivated and dysregulated in KO GFAP^+^ cells (Figure 8B). The canonical Wnt pathway is directly associated with the cell cycle by modulating the expression of cyclins D1, D2, and c-Myc (Baek et al., 2003; Da Silva et al., 2021). Therefore, we examined the cell cycle regulatory genes and found that TSK deficiency severely affected their expression. We specifically observed that the key cell cycle-promoting cyclin genes encoding cyclin D, cyclin E, and cyclin A were misspliced and had increased expression for cyclin D and cyclin A (Figures 8C,D). Conversely, the key cell cycle regulators *Cdk2*, *Cdk4/6*, *Cdc25A*, *Mizl*, *Scf*, and *Apc* had either decreased expression, or were misspliced, or both (Figure 8D; Supplementary Table S7; Supplementary Material S2). Pathway analysis showed that such dysregulation affected the overall cell cycle, potentially resulting in the uncontrolled proliferation of cells, mimicking a cancer-like state; we detected the similar enrichment categories in the pathway enrichment network analysis of DEGs. We also found significant enrichment in circadian rhythm genes among DEGs with decreased expression. Circadian rhythms and cell cycles are interrelated processes. In particular, we found that the expression of circadian rhythm regulators, such as *Clock, Per1, Per2, Bhlhe40, Nr1d1,* and *Prkab1* was decreased (Supplementary Table S7; Supplementary Material S2, 3). These genes are known to determine the timing of cell cycle reentry and the maintenance of proliferative homeostasis. Therefore, the decreased expression of these regulatory genes dysregulates the cell cycle processes towards uncontrolled proliferation or apoptosis (Farshadi et al., 2020). Thus, TSK deficiency resulted in transcriptional and splicing dysregulation of the Wnt signaling and underlying cell cycle in GFAP^+^ NSCs.

### Tsukushi deficiency impaired the sonic hedgehog and mTOR pathways

**FIGURE 9:**
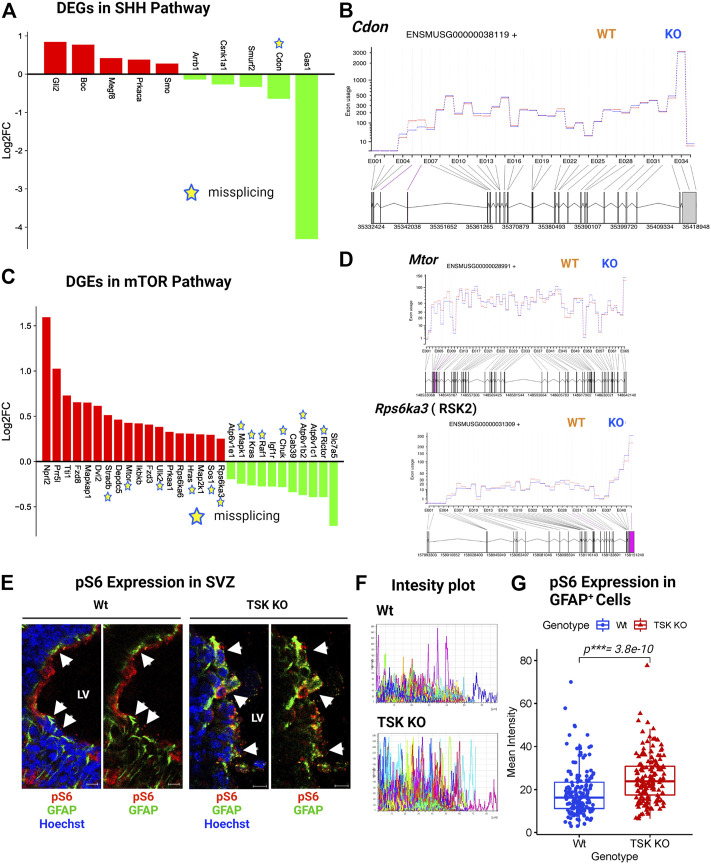
TSK regulates the ciliogenesis-associated SHH and mTOR signaling. **(A)** Expression of SHH pathway-associated DEGs. Red and green bars indicate genes with increased and decreased expression, respectively **(B)** Fitted splicing plot for DEU in representative SHH pathway-associated gene *Cdon*. Pink indicates the exon with significant differential exon usage. **(C)** Expression of DEGs associated with the mTOR pathway. Red and green bars indicate genes with increased and decreased expression, respectively. Expression bars are annotated similar to those in (A). **(D)** Fitted splicing plot for DEU in representative mTOR pathway-associated crucial gene *Mtor* and *Rps6ka3*. Plot is annotated similar to that in (B). **(E)** Representing images of P3 stage TSK KO SVZ immunostained for GFAP (Green), pS6 (Red) and Nuclei (Hoechst, blue). Arrowheads indicates pS6 expression in GFAP^+^ cells. Scale bar = 10 μm. **(F)** Representative fluorescent (Alexa flour 647) intensity plot for pS6 expression in GFAP^+^ cells of a single SVZ. Colored curves represent individual cells. The peak height of the curve reflects the intensity value, while the curve length reflects the measured area. **(G)** Mean intensity distribution of pS6 expression in GFAP^+^ cells SVZ. Distribution is shown by box plot with jitters. Each dot represents an individual cell. Number of brain samples: nTSK KO = 3, nWt = 3.

In TSK KO mice, ciliogenesis is disrupted, resulting in a dispersed distribution of ciliary bundles (Ito et al., 2021). Along with the Wnt, we found that KO GFAP^+^ cells were associated with DEGs involved in ciliogenesis and stemness-associated pathway, such as hedgehog and mTOR. These signaling pathways play critical roles in stem cell maintenance, tissue patterning, and ciliogenesis (Sancak et al., 2010; Lipton and Sahin, 2014; Akhshi and Trimble, 2021; Da Silva et al., 2021). Sonic hedgehog (SHH) signaling is required for ciliogenesis and patterning of neuroepithelial cells (Akhshi and Trimble, 2021). More specifically, *Shh*, *Gli*, *Ptch*, *Smo*, and *Gli* all play important roles in SHH signaling. Briefly, SHH binds to PTCH and activates SMO, which then translocates to the primary cilium and represses suppressor of fused (SUFU), activating GLI. In turn, GLI controls the expression of genes that regulate SHH signaling, cell proliferation, and apoptosis (Park et al., 2019). In particular, we detected the upregulation of *Gli* and *Smo* in KO mice (Figure 9A). The upregulation of *Smo* might have triggered the downstream activation of the SHH regulatory gene *Gli2* in KO cells, resulting in the overall activation of the SHH pathway. However, we observed missplicing in the putative SHH coreceptor *Cdon* with decreased expression (Figures 9A,B). In addition, we found that other non-DEG SHH-associated genes (*Smurf1*, *Kif3a*, *Spop*, *Ccnd1*, *Bcl2*, etc.) were also misspliced, suggesting dysregulated SHH signaling in TSK deficient GFAP^+^ cells (Supplementary Material S2, 3).

mTOR signaling, which is mediated by the serine/threonine kinase mTOR, is involved in neurogenesis, neuronal differentiation, migration, and brain development (LiCausi and Hartman, 2018). Moreover, mTOR is directly involved in lipid synthesis, protein translation, and cell autophagy (Park et al., 2019). Pathological conditions, such as neurological diseases, cancer, and metabolic problems, have been associated with dysfunctions in the mTOR signaling (Lipton and Sahin, 2014). The gene expression patterns suggested an activated mTOR pathway in KO GFAP^+^ cells through the increased expression of essential genes (*Mtor*, *Dvl2*, *Fdz8*, *Ikbkb*, etc.) and decreased expression of regulatory genes (*Kras*, *Raf1*, *Igf1r*, *Ricor*, etc.) (Figure 9C). However, we noticed that 12 out of the 29 DEGs in mTOR signaling were aberrantly spliced, with the crucial gene *Mtor* (Figures 9C,D; Supplementary Table S7). mTOR signaling activates S6 kinase (encoded by *RSK*s) that phosphorylate Rps6 (pS6) and regulate the SVZ NSC proliferation (Paliouras et al., 2012; Hartman et al., 2013). KO group also showed significantly upregulated and misspliced RSK2 encoding gene (Figure 9D; Supplementary Material S2). In order to assess the effect of overexpressed misspliced mTOR pathway genes, we compared the expression of phosphorylated Rps6 (pS6) in Wt and TSK KO SVZ GFAP^+^ cells. We detected significant (p***= 3.8e-10) overexpression of pS6 in the KO group (Figures 9E–G). These data suggest that the misspliced overactivated component of the mTOR pathway may also be responsible for impairing SVZ GFAP^+^ NSC survival.

## Discussion

The integrity of the brain stem/progenitor cells demands the stringent regulation of critical signaling processes and a coordinated posttranscriptional regulation to ensure appropriate organ development. Here, we showed that Wnt signaling, cell cycle and mTOR pathways were overactivated, whereas the circadian rhythm and hedgehog signaling pathways were negatively affected in KO GFAP^+^ cells. Surprisingly, TSK deficiency disrupted the vital posttranscriptional regulation of RNA splicing and increased the transcript burden. Consequently, missplicing was evident in all DEG-associated pathways. Thus, our findings demonstrated that TSK not only influences key developmental pathways in GFAP-positive cells but also maintains transcript diversity through appropriate splicing. In particular, dysregulated and incorrectly spliced genes of critical signaling pathways, such as Wnt, cell cycle, mTOR, and SHH can have a devastating effect on the fate of LV GFAP^+^ stem/progenitor cells, compromising lineage survival and contributing to aberrant LV morphogenesis.

Alternate splicing is a critical process for maintaining the levels and diversity of the cell’s transcriptome and proteome. Compared with other tissues, alternate splicing is more conserved in the brain (Raj and Blencowe, 2015). It is a tightly regulated process that participates in every step of brain development, including cell fate determination, neurogenesis, axon guidance, synaptogenesis, and neuronal migration. Alternate splicing of key transcriptional regulators or epigenetic factors reprograms the transcriptome and participates in stem cell fate determination (Su et al., 2018; Weyn-Vanhentenryck et al., 2018). In this study, we discovered that TSK deficiency significantly disrupted the splicing process of LV GFAP^+^ cells, resulting in genome-wide misspliced DEU genes that affect fate-determining developmental signaling and key transcriptional regulatory processes, thus leading to an aberrant cell fate. DEU has been associated with neuronal migration, synaptogenesis defects, and Alzheimer’s disease (Beffert et al., 2005; Yano et al., 2010, 2; Hinrich et al., 2016). For instance, knocking out *Ptbp1* resulted in differential exon use in *Flna* and *Flnb*, which participate in altering neural progenitor cell fate and developing hydrocephalus (Zhang et al., 2016). TSK dysfunction can result in hydrocephalus in both mice and humans, and our research suggested that aberrant splicing in LV GFAP^+^ stem/progenitor cells contributed to disease progression. More specifically, TSK deficiency altered both the splicing and transcriptome levels of key developmental signaling genes, including *Wnt*, *Shh*, *mTor*, hippo, *FoxO*, *Notch*, and *Mapk*. Wnt signaling is highly expressed during perinatal development in mice, affecting cell fate, proliferation, and transcription (Baek et al., 2003; Da Silva et al., 2021). For instance, deficiency of the Wnt receptor Frizzled3 causes LV enlargement in mice (Stuebner et al., 2010, 3). TSK binds to the Frizzled3 receptor and inhibits Wnt, thus regulating the entire Wnt signaling process (Ito et al., 2021). Consequently, our study identified several DEGs in Wnt signaling, indicating its overactivation through the increased expression of important genes (encoding Frizzled, DVL, and cyclin D) and decreased expression of regulatory genes (encoding axin, APC, and DKK). However, the major genes of the canonical Wnt pathway (encoding WNT, WNT2b, β-catenin, and cyclin D) and its regulators (encoding axin and APC) exhibited DEU. Similarly, the *Daam1* and *Jnk* transcriptional regulators in the planar cell polarity pathway exhibited DEU. Hence, dysregulation of Wnt signaling in the KO SVZ may have resulted from loss of the multifunctional role of TSK, while lack of extracellular TSK caused increased expression of receptor and cell cycle genes, and lack of nuclear TSK caused changes in associated gene splicing. Our data suggest that TSK might work on these cells in autocrine fashion to regulate Wnt signaling. However, to pinpoint the exact extracellular signaling nature of TSK in SVZ, a thorough analysis from embryonic NSCs is necessary. In KO mouse LV cells, the proliferation was increased at P0, whereas apoptosis was increased at P10 (Ito et al., 2021). Disruption of the Wnt signaling has direct effects on cell cycle. For instance, we observed a dysregulated cell cycle process in KO GFAP^+^ cells as a result of cyclin dysregulation. Besides cell cycle regulation, cyclins also influence transcription, cell differentiation, and cell death (Hydbring et al., 2016). Dysregulation of cyclins has been linked to the development of neurodegenerative disorders (Seo and Park, 2020). Both the expression of cyclin D and cyclin A were increased in KO GFAP^+^ cells, whereas the expression of cyclin E was decreased; moreover, all cyclins displayed DEU. Consequently, dysregulation of cyclins in LV stem/progenitor cells by TSK-deficiency might have contributed to the early overactivation of proliferation processes, leading to an increase in cell death later in development.

Moreover, TSK deficiency affected the mTOR pathway by increasing the expression of essential genes (*Mtor*, *Tti1*, *Prr5*, *Ikbkb*, etc.) and decreasing that of regulatory genes (Atp6v1c1, Rictor, Raf1, Kras, etc.). As a result, overactivation of the mTOR signaling may have triggered an increase in pS6 expression in the TSK KO GFAP + cells. The mTOR-pS6 pathway is necessary for NSC self-renewal in the SVZ (LiCausi and Hartman, 2018). Overactivation of pS6 may have disrupted the delicate balance of the signaling process, along with other dysregulated pathways contributing to the exhaustion of SVZ GFAP^+^ NSCs in TSK KO hydrocephalic mouse model. In addition to cyclins and mTOR, circadian rhythms might also have contributed to the abnormal proliferation of LV cells in KO mice. Circadian clock genes govern cell cycle checkpoints, thereby regulating cell cycle (Farshadi et al., 2020). In KO GFAP^+^ cells, the expression of regulatory components of the process, such as clock and period genes, was decreased, indicating a dysfunction in oscillatory control. TSK-deficiency affects ciliogenesis in the LV wall, resulting in a scattered ciliary distribution (Ito et al., 2021). Deficiency in LV cilia disrupts the absorption, metabolism, and movement of cerebrospinal fluid, leading to hydrocephalus (Feldner et al., 2017; Takagishi et al., 2017; Sakamoto et al., 2021). During early postnatal development, GFAP^+^ cells include ependymal or ependymal progenitor cells that express TSK. The expression of FOXJ1, a transcription factor that modulates ciliogenesis, was found to be increased in the absence of TSK. Wnt, SHH, and mTOR signaling participate in ciliogenesis. In our study, we found that KO GFAP^+^ stem/progenitor cells have dysregulated SHH and mTOR signaling-like gene expression patterns. During ciliogenesis, SHH activates SMO *via* a noncanonical route (Akhshi and Trimble, 2021). We observed a number of important SHH signaling genes in KO GFAP^+^ cells in our study. The levels of expression of both *Smo* and *Gli2* were increased, whereas those of *Smurf2, Gas1,* and *Cdon* were decreased, and associated with DEU in *Cdon*, indicating that TSK might be involved in the activation of the SHH pathway. The aberrant ciliogenesis observed in the LV wall of hydrocephalic KO mice might have been caused by the overactivation of Wnt, SHH, and mTOR, which were dysregulated as a consequence of splicing errors. However, the mechanism by which TSK interacts with the SHH or mTOR pathways remains unknown. It is plausible that interference with Wnt might alter SHH or mTOR signaling, or that TSK might directly interact with SHH and mTOR; additional research is thus required.

**FIGURE 10:**
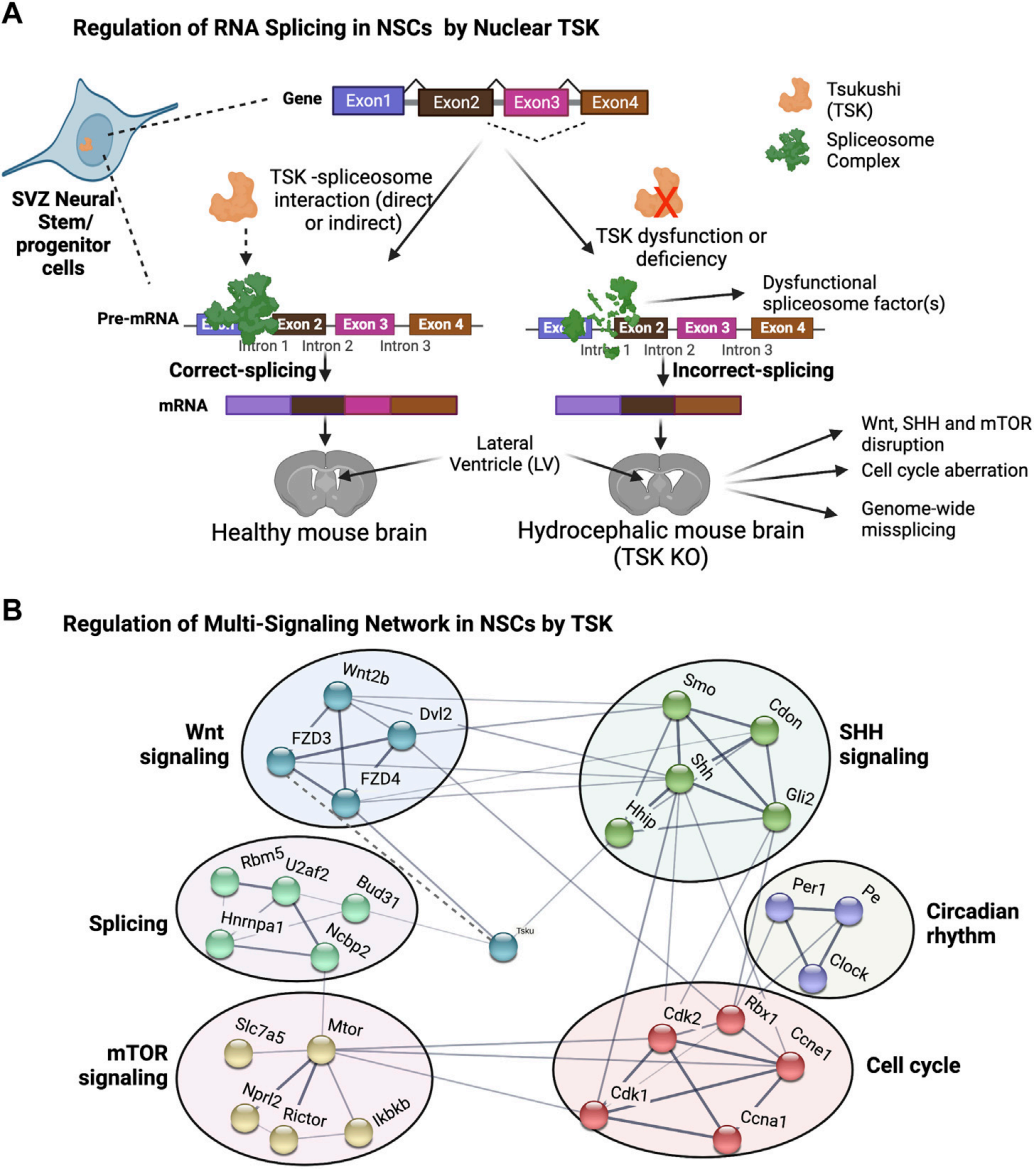
Proposed model for TSK in splicing and singling regulation in hydrocephalus. **(A)** Functional TSK expressed by stem/progenitor cells interacts with the spliceosome and ensures that pre-mRNA splicing is properly regulated, maintaining proper signaling and cell fate. Tsukushi deficiency or dysfunction, on the other hand, results in spliceosomal gene dysregulation, leading to the improper spliceosomal complex formation and missplicing of pre-mRNA. Accordingly, the loss of correct splicing affects the protein function and impairs the signaling process, causing LV enlargement by altering the NSCs. **(B)** String-based analysis of protein-protein interactions of TSK with development associated multi-signaling components. The nodes represent proteins. Colors of the nodes represent the cluster groups (K-mean clustering). The thickness of the edges (solid line) is proportional to their confidence scores (Minimum confidence score for the interaction = 0.4). The dotted line indicates that the interaction is not yet included in the string database and evident in our previous publication (Ito et al., 2021).

Surprisingly, we found that TSK deficiency drastically affected the splicing and expression of spliceosome-encoding genes. The gene with the highest number of differential exons (14 exons) was *Rbm5*, which is a component of the RNA-binding spliceosome. We identified a significant enrichment of all misspliced genes with decreased expression related to posttranscriptional regulation *via* direct or indirect binding to mRNA, indicating that TSK is essential for the regulation of posttranscriptional regulators. Both the decreased expression and missplicing of spliceosome component genes (*Snrnp70*, *Hnmpc*, *Dhx15*, etc.) and increased expression of misspliced component genes (*Srsf5*, *Puf60*, and *Dhx16*) were evident in KO cells. These differentially expressed or misspliced genes are known to considerably affect splicing processes. For instance, UAP56 is required for initial spliceosome formation, PRP18 stabilizes the exon and spliceosome components throughout the second step of pre-mRNA processing, and PRP43 participates in the disassembly of the spliceosome complex following splicing (Crotti et al., 2007; Shen et al., 2007; Fourmann et al., 2017). The expression of all these genes was decreased when *Prp43* and *Prp18* were misspliced in KO GFAP^+^ cells. In addition, *Brr2* and *Smul14*, which are involved in spliceosome activation, and *Prp2*, which initiates the initial step of spliceosome-RNA interaction, were also misspliced. Consequently, TSK deficiency affects the assembly, reassembly, and pre-mRNA binding of the spliceosome complex, which might be the cause of the genome-wide differential exon use that disrupts essential developmental processes. In the prediction of protein-protein interactions, we noticed that mouse TSK might interact with the BUD31 spliceosome component and hence engage in the splicing process. This is because the mouse TSK has putative similarity to the LEA1 protein of *Saccharomyces cerevisiae*. However, to confirm this claim, research using rigorous protein-protein interaction experiments, such as coimmunoprecipitation, is required. In the DEU of whole LV cells, we observed that TSK deficiency strongly affected the splicing of spliceosome and ribosomal genes, but not that of developmental genes. Hence, the effect of TSK-deficiency on splicing dysregulation is cell type-specific and TSK may regulate the splicing of spliceosome genes generally among LV cells. Furthermore, we found that TSK-deficiency also triggered missplicing in transcriptional and signaling associated genes in other organ (muscle tissue) as well (Figures 6E–G; Supplementary Table S6). Thus, the results suggest that TSK has a universal function in maintaining cellular splicing. TSK is known to participate in extracellular signaling as a secretory protein (Ito et al., 2021; Istiaq and Ohta, 2022). Nonetheless, we detected TSK expression in the nucleus of GFAP^+^ cells. Hence, our research indicates that TSK might have an additional nuclear function in posttranslational regulation. Human TSK is also localized both in the nucleus, as it is in mice (Sjöstedt et al., 2020). We posit that nuclear TSK ensures correct splicing and transcriptome integrity and, in combination with extracellular TSK (Wnt inhibitor), regulates NSCs’ fate, thus protecting the LV architecture (Figures 10A,B). Future studies need to determine whether the administration of TSK in the LV can rescue the abnormal splicing while preventing LV expansion in hydrocephalic murine brains.

In summary, our study uncovered a previously unknown function of TSK in the regulation of splicing. Disruption of splicing and consequent major developmental pathways in GFAP^+^ cells of KO mice suggest that TSK is essential for posttranscriptional control and signaling modulation in SVZ niche during LV development. To our knowledge, although the abnormal splicing of certain genes, such as *L1cam*, *C11orf70*, and *Lrrc48* has been linked to the development of hydrocephalus, a genome-wide splicing anomaly has not been previously reported (Rosenthal et al., 1992; Ferese et al., 2016). The role of TSK, in modulating splicing and developmental signaling mediators in GFAP^+^ stem/progenitor cells, offers crucial understanding into aberrant LV development during hydrocephalus.

## Data Availability

All the processed data needed to evaluate the findings in the paper are present in the paper and/or the Supplementary Materials. Ultra-low input RNA-seq raw data from SVZ GFAP-expressing cells were deposited in the Gene Expression Omnibus (GEO) database (https://www.ncbi.nlm.nih.gov/geo/) under the accession no: GSE205788. The raw data is not publicly available yet; however, access can be granted from the corresponding author after reasonable request. Bulk RNA-seq data from SVZ tissue is available in the DDBJ Sequence Read Archive (https://www.ddbj.nig.ac.jp/dra/index-e.html) under the accession number DRA008517. Bulk RNA-seq data from muscle tissue is publicly available from GEO database (Accession no: GSE193089).

## Supplementary material

Supplementary Video S1 TSK localization in the SVZ GFAP^+^-SOX^+^ expressing cells. The video represents the TSK localization analysis in Figure 2D. Gray represents the nuclei, orange gradient (glow) represents TSK, and green indicates GFAP.

## Nomenclature

### Abbreviations

Wt: Wild type mice (TSK^+/+^)
WT: GFAP^GFP^ expressing wild type mice (GFAP^GFP/+^/TSK^+/+^)
TSK KO: Knock-out mice (TSK^-/-^)
KO: GFAP^GFP^ expressing TSK knock-out mice (GFAP^GFP/+^/TSK^-/-^)
GFAP^+^: Glial fibrillary acidic protein expressing cells
SOX2^+^: SRY-box transcription factor 2 expressing cells
S100β^+^: S100 calcium binding protein B expressing cells
DET: Differentially expressed transcript
DEG: Differentially expressed gene
DEU: Differential exon usage
LV: Lateral ventricle
