# Supplementary material for: Tsukushi proteoglycan maintains RNA splicing and developmental signaling network in GFAP-expressing subventricular zone neural stem/progenitor cells
Source: Front Cell Dev Biol. 2022 Nov 21;10:994588. doi: 10.3389/fcell.2022.994588 (PMC9720143; doi:10.3389/fcell.2022.994588)
Supplement: Supplementary file 4 [file DataSheet1.PDF]

Supplementary Materials for

Tsukushi proteoglycan maintains RNA splicing and developmental signaling in GFAP-expressing subventricular zone neural stem or progenitor cells

Arif Istiaq, Terumasa Umemoto, Naofumi Ito, Toshio Suda, Kenji Shimamura, Kunimasa Ohta*
*Corresponding author. Email: ohta9203@artsci.kyushu-u.ac.jp

This PDF includes:

Fig. S1. Representative secondary antibody control images for the immunofluorescence analysis.
Fig. S2. Per base sequence quality of the FASTQ files of each sample.
Fig. S3. Transcript estimation statistics for SVZ GFAP+ cells.
Fig. S4. Single cell RNA-seq analysis of *Tsku* expression in various cell types in adult murine SVZ.
Table S1. FACS analysis of GFAP^{GFP} expressing cells in SVZ samples.
Table S2. Pathway network enrichment analysis (KEGG) for DEGs in SVZ GFAP+ cells.
Table S3. Pathway network enrichment analysis for DEGs that have DEU in SVZ GFAP+ cells.
Table S4. Pathway network enrichment analysis for DEU genes at TSK KO SVZ tissue.
Table S5. Estimation of protein-protein interaction network for DEU genes in TSK KO SVZ tissue.
Table S6. Pathway network enrichment analysis for DEU genes in TSK KO muscle tissue.
Table S7. Key pathway associated DEGs in SVZ GFAP expressing cells.
Legends for:
Data file S1. Estimation of significant DEGs in SVZ GFAP+ cells by Ballgawn.
Data file S2. Significant DEGs that have DEUs in GFAP+ cells.
Data file S3. Significant DEU genes with chromosomal location and number of exon changing events in SVZ GFAP+ expressing cells.
Data file S4. Significant DEU genes with chromosomal location and number of exon changing events in SVZ tissue.
Data file S5. Significant DEU genes with chromosomal location and number of exon changing events in muscle tissue.
Data file S6. String-based protein-protein interaction (PPI) analysis for TSK and spliceosome associated factors that have DEU.

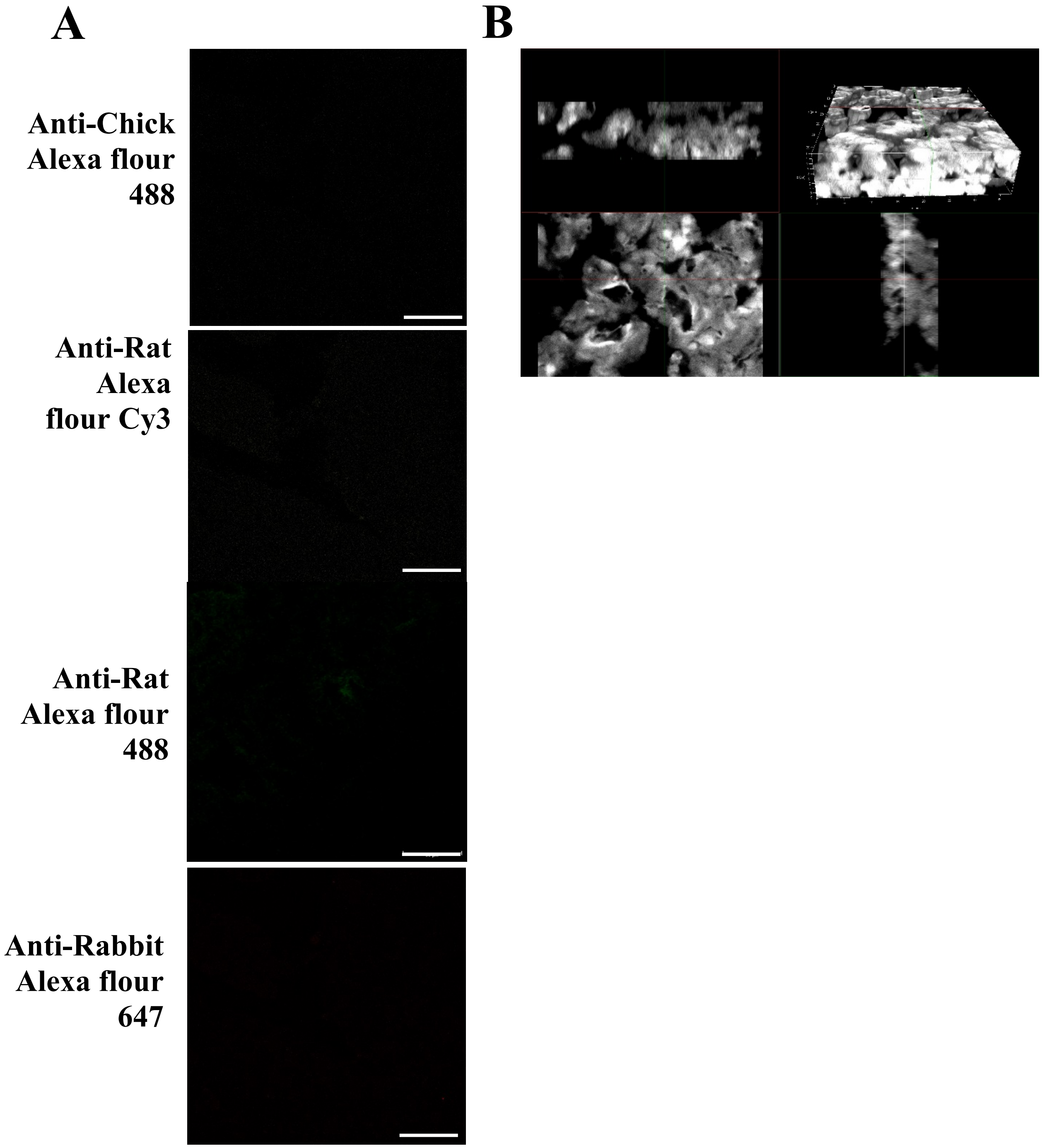

Fig. S1. Representative secondary antibody control images for the immunofluorescence analysis. Scale Bar 50 um. (**A**) Analysis of secondary antibody non-specific staining. Background was not detected or was faint (anti-Rat 488). (B) Z-stack 3D analysis of secondary antibody background staining. Control images were obtained under the same imaging conditions to ensure equal pixel size. The control images were all taken focusing on the SVZ area.

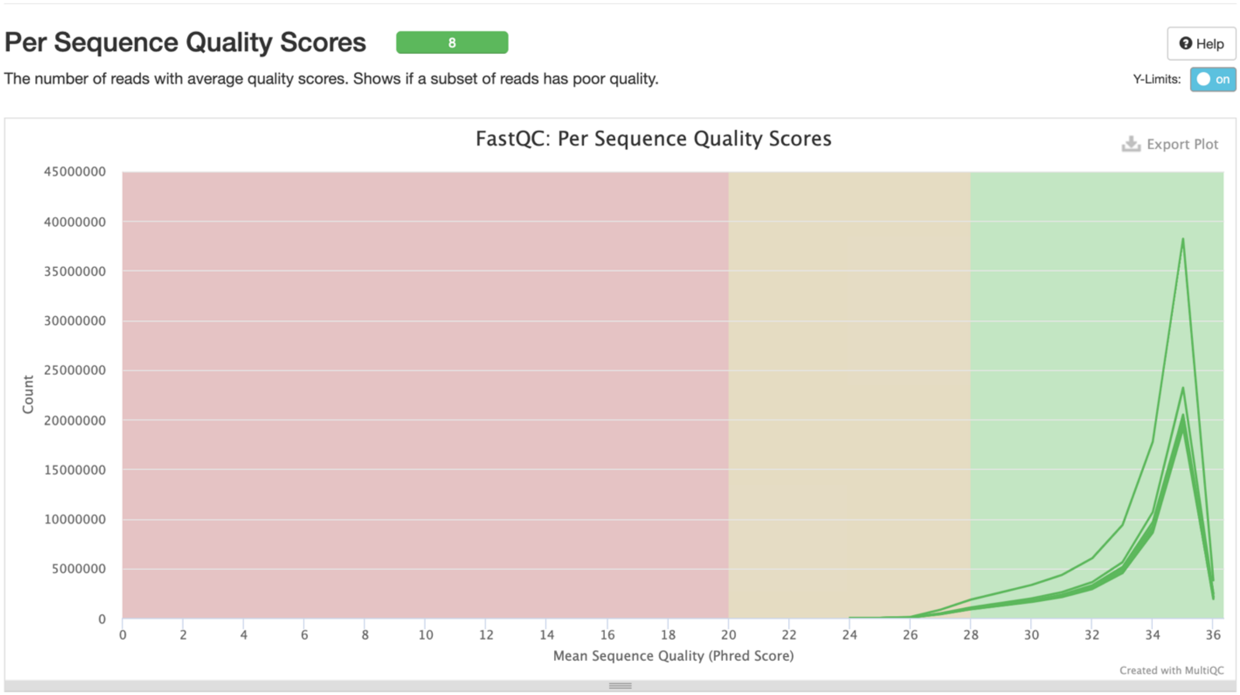

Fig. S2. **Per base sequence quality of the FASTQ files of each sample.** Green curve represents sequence read quality for an individual brain sample (Fastq).

A

B

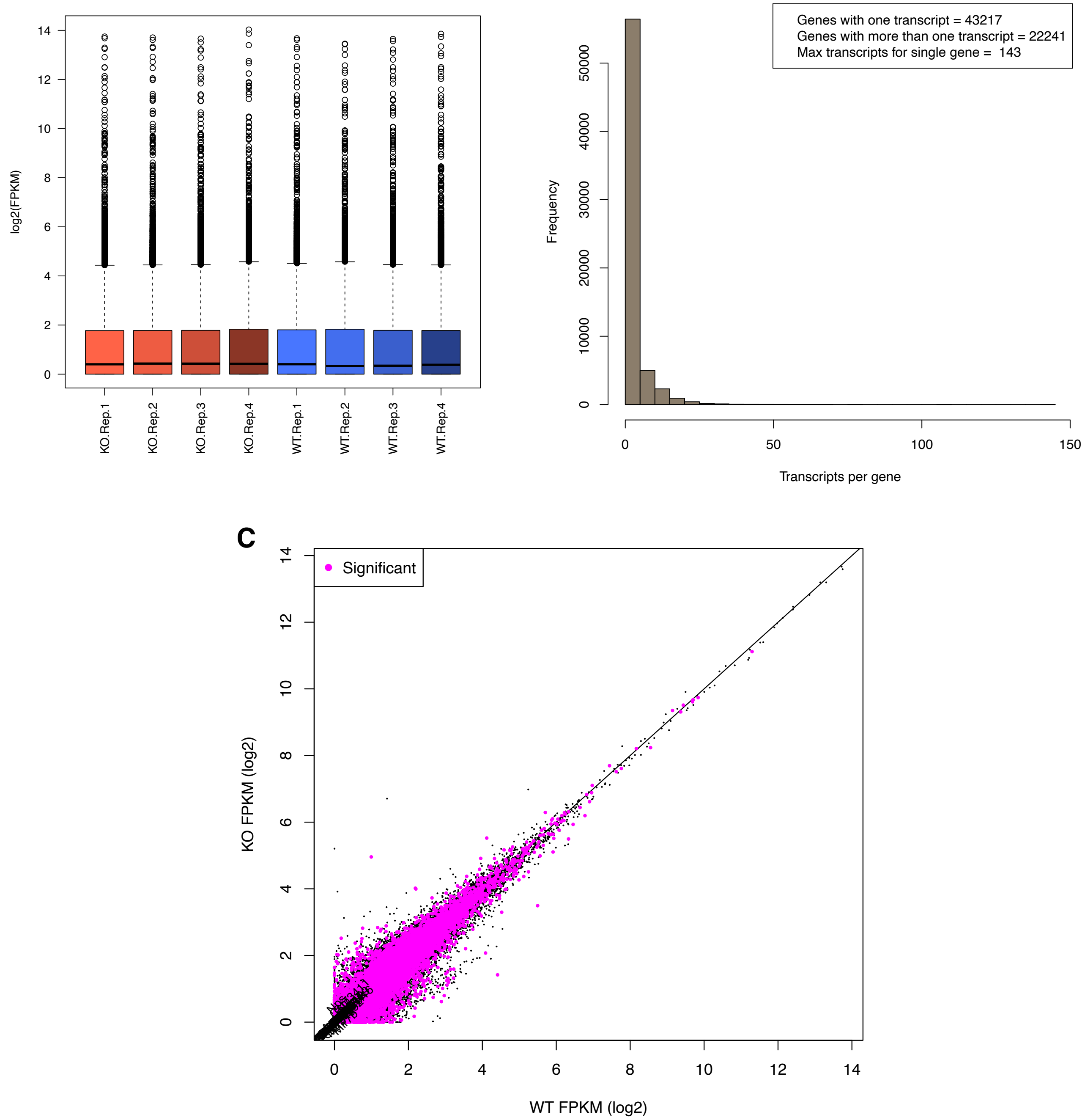

Fig. S3. Transcript estimation statistics for SVZ GFAP-expressing cells. (**A**) Fragments per kilobase of exon per million mapped fragments (FPKMs0 distribution in all 8 libraries from the WT (n=4) and KO (n=4) samples. Color of the boxes corresponds to the samples (**B**) Distribution of transcript counts per gene. (**C**) Significant DEGs between WT and KO group. Pink dots represent significant genes.

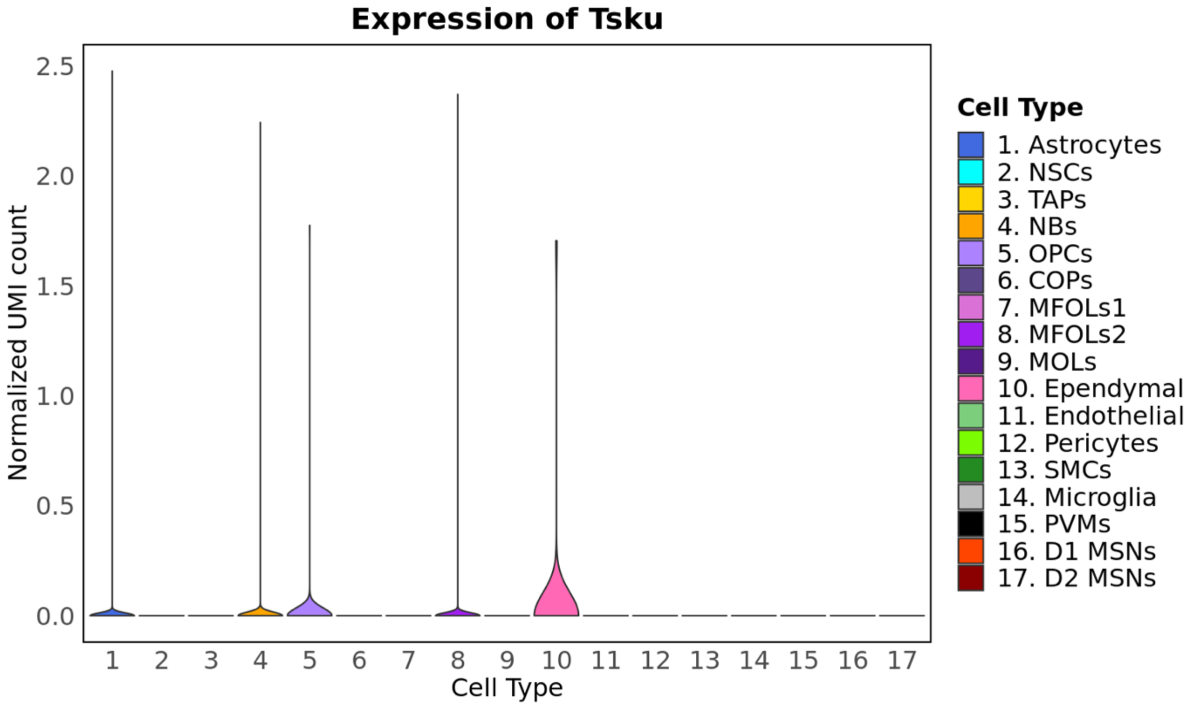

Fig S4. Single cell RNA-seq analysis of *Tsku* expression in various cell types in adult murine SVZ (Zywitza et al., 2018). NSCs: neural stem cells; TAPs: transient amplifying progenitors; NBs: neuroblasts; OPCs: oligodendrocyte progenitor cells; COPs: differentiation-committed oligodendrocyte precursors; MFOLs: myelin forming oligodendrocytes; MOLs: mature oligodendrocytes; SMCs: smooth muscle cells; PVMs: perivascular macrophages; MSNs: medium spiny neurons.

Table S1. FACS analysis of $GFAP^{GFP}$ expressing cells in SVZ samples

Sample	GFP positive cells (percentage)	Standard deviation
WT. Rep.1	5.49	4.52544933
WT. Rep.2	5.36	
WT. Rep.3	13.9	
WT.Rep.4	12.5	
KO. Rep.1	7.57	1.45409766
KO. Rep.2	5.47	
KO. Rep3	8.17	
KO. Rep4	5.31	

Table S2. Pathway network enrichment analysis (KEGG) for DEGs in SVZ $GFAP^+$ cells. Pathway enrichments were estimated for KEGG. FDR = False discovery rate.

PATHWAY	TOTAL	EXPECTED	HITS	P.VALUE	FDR
PROTEIN PROCESSING IN ENDOPLASMIC RETICULUM	163	16.1	45	7.21E-11	2.26E-08
METABOLIC PATHWAYS	1490	147	212	1.69E-09	2.65E-07
NEUROTROPHIN SIGNALING PATHWAY	121	11.9	28	1.28E-05	0.00128
OOCYTE MEIOSIS	116	11.4	27	1.62E-05	0.00128
UBIQUITIN MEDIATED PROTEOLYSIS	139	13.7	30	2.7E-05	0.00149
TERPENOID BACKBONE BIOSYNTHESIS	23	2.27	10	2.85E-05	0.00149
ADHERENS JUNCTION	72	7.1	19	4.69E-05	0.0021
SPLICEOSOME	133	13.1	28	7.91E-05	0.00311
LONG-TERM POTENTIATION	67	6.61	17	0.000196	0.00684
PROTEASOME	46	4.54	13	0.000352	0.0111
CIRCADIAN RHYTHM	30	2.96	10	0.000394	0.0112
HEPATITIS B	163	16.1	30	0.000543	0.0131
CARBON METABOLISM	120	11.8	24	0.000558	0.0131
HUNTINGTON'S DISEASE	194	19.1	34	0.000607	0.0131

NUCLEOTIDE EXCISION REPAIR	43	4.24	12	0.000669	0.0131
VASOPRESSIN-REGULATED WATER REABSORPTION	43	4.24	12	0.000669	0.0131
RENAL CELL CARCINOMA	68	6.71	16	0.000744	0.0134
GLUCAGON SIGNALING PATHWAY	102	10.1	21	0.000817	0.0134
VALINE, LEUCINE AND ISOLEUCINE DEGRADATION	56	5.52	14	0.000828	0.0134
PATHWAYS IN CANCER	535	52.8	75	0.00092	0.0134
MITOPHAGY - ANIMAL	63	6.21	15	0.000946	0.0134
CHRONIC MYELOID LEUKEMIA	76	7.49	17	0.000952	0.0134
FATTY ACID METABOLISM	57	5.62	14	0.001	0.0134
PROGESTERONE-MEDIATED OOCYTE MATURATION	90	8.88	19	0.00104	0.0134
INSULIN SIGNALING PATHWAY	140	13.8	26	0.00108	0.0134
MTOR SIGNALING PATHWAY	155	15.3	28	0.00111	0.0134
PHOSPHATIDYLINOSITOL SIGNALING SYSTEM	98	9.66	20	0.00121	0.0141
FRUCTOSE AND MANNOSE METABOLISM	35	3.45	10	0.00153	0.0171
INOSITOL PHOSPHATE METABOLISM	73	7.2	16	0.00167	0.018
TIGHT JUNCTION	167	16.5	29	0.00172	0.018
GLYCOSYLPHOSPHATIDYLINOSITOL (GPI)-ANCHOR BIOSYNTHESIS	25	2.47	8	0.00203	0.0206
AMPK SIGNALING PATHWAY	126	12.4	23	0.00259	0.0249
GNRH SIGNALING PATHWAY	90	8.88	18	0.00263	0.0249
ESTROGEN SIGNALING PATHWAY	134	13.2	24	0.00274	0.0249
N-GLYCAN BIOSYNTHESIS	50	4.93	12	0.00278	0.0249
CITRATE CYCLE (TCA CYCLE)	32	3.16	9	0.00294	0.0256
CENTRAL CARBON METABOLISM IN CANCER	64	6.31	14	0.00325	0.0276

BIOSYNTHESIS OF AMINO ACIDS	78	7.69	16	0.00342	0.0282
CELL CYCLE	123	12.1	22	0.0041	0.033
HIPPO SIGNALING PATHWAY	154	15.2	26	0.00431	0.0334
APELIN SIGNALING PATHWAY	139	13.7	24	0.00446	0.0334
PROTEIN EXPORT	28	2.76	8	0.00447	0.0334
COLORECTAL CANCER	88	8.68	17	0.00496	0.0362
ENDOCYTOSIS	270	26.6	40	0.00554	0.0395
PROSTATE CANCER	97	9.57	18	0.00606	0.0423
ERBB SIGNALING PATHWAY	84	8.28	16	0.00726	0.0485
PEROXISOME	84	8.28	16	0.00726	0.0485
CHOLINE METABOLISM IN CANCER	99	9.76	18	0.00752	0.0492
STEROID BIOSYNTHESIS	19	1.87	6	0.00796	0.051
FATTY ACID DEGRADATION	50	4.93	11	0.00827	0.0519
GLUTATHIONE METABOLISM	64	6.31	13	0.00862	0.0531
FOXO SIGNALING PATHWAY	132	13	22	0.00958	0.0578
THYROID HORMONE SYNTHESIS	73	7.2	14	0.0109	0.0648
BACTERIAL INVASION OF EPITHELIAL CELLS	74	7.3	14	0.0123	0.0716
GLYCEROPHOSPHOLIPID METABOLISM	97	9.57	17	0.0132	0.0735
WNT SIGNALING PATHWAY	160	15.8	25	0.0134	0.0735
HIF-1 SIGNALING PATHWAY	105	10.4	18	0.0137	0.0735
PANCREATIC CANCER	75	7.4	14	0.0138	0.0735
MAPK SIGNALING PATHWAY	294	29	41	0.0138	0.0735
PHOSPHONATE AND PHOSPHINATE METABOLISM	6	0.592	3	0.0152	0.0796
AUTOPHAGY - ANIMAL	130	12.8	21	0.0157	0.0809

ACUTE MYELOID LEUKEMIA	69	6.8	13	0.0161	0.0813
HOMOLOGOUS RECOMBINATION	41	4.04	9	0.0164	0.0819
THYROID HORMONE SIGNALING PATHWAY	115	11.3	19	0.0168	0.0822
MELANOGENESIS	100	9.86	17	0.0176	0.0851
VIRAL CARCINOGENESIS	231	22.8	33	0.0184	0.0877
DOPAMINERGIC SYNAPSE	135	13.3	21	0.0232	0.109
ENDOMETRIAL CANCER	58	5.72	11	0.0245	0.113
PARKINSON'S DISEASE	144	14.2	22	0.0248	0.113
BASAL TRANSCRIPTION FACTORS	44	4.34	9	0.0255	0.113
HEDGEHOG SIGNALING PATHWAY	44	4.34	9	0.0255	0.113
GLYOXYLATE AND DICARBOXYLATE METABOLISM	31	3.06	7	0.0283	0.124
RIG-I-LIKE RECEPTOR SIGNALING PATHWAY	68	6.71	12	0.0324	0.137
ADRENERGIC SIGNALING IN CARDIOMYOCYTES	148	14.6	22	0.0327	0.137
2-OXOCARBOXYLIC ACID METABOLISM	19	1.87	5	0.0332	0.137
BIOSYNTHESIS OF UNSATURATED FATTY ACIDS	32	3.16	7	0.0333	0.137
LONG-TERM DEPRESSION	61	6.02	11	0.0344	0.14
ENDOCRINE RESISTANCE	93	9.17	15	0.0378	0.152
LONGEVITY REGULATING PATHWAY - MULTIPLE SPECIES	62	6.11	11	0.0382	0.152
SALMONELLA INFECTION	78	7.69	13	0.0405	0.159
GLYCOSAMINOGLYCAN BIOSYNTHESIS - KERATAN SULFATE	14	1.38	4	0.0421	0.163
PROTEOGLYCANS IN CANCER	204	20.1	28	0.0443	0.17
SIGNALING PATHWAYS REGULATING PLURIPOTENCY OF STEM CELLS	137	13.5	20	0.0474	0.176
NOTCH SIGNALING PATHWAY	49	4.83	9	0.0477	0.176
MRNA SURVEILLANCE PATHWAY	96	9.47	15	0.0482	0.176
EGFR TYROSINE KINASE INHIBITOR RESISTANCE	80	7.89	13	0.0483	0.176

Table S3. Pathway network enrichment analysis (KEGG) for DEGs that have DEU in SVZ GFAP^{+} cells. Pathway enrichments were estimated for KEGG. FDR = False discovery rate.

PATHWAY	TOTAL	EXPECTED	HITS	P.VALUE	FDR
SPLICEOSOME	133	4.48	16	9.16E-06	0.00288
OOCYTE MEIOSIS	116	3.9	14	3.22E-05	0.00505
TERPENOID BACKBONE BIOSYNTHESIS	23	0.774	6	8.58E-05	0.00898
ENDOMETRIAL CANCER	58	1.95	9	0.000122	0.00952
INOSITOL PHOSPHATE METABOLISM	73	2.46	10	0.000152	0.00952
PROGESTERONE-MEDIATED OOCYTE MATURATION	90	3.03	11	0.000202	0.0104
UBIQUITIN MEDIATED PROTEOLYSIS	139	4.68	14	0.000233	0.0104
PROTEIN PROCESSING IN ENDOPLASMIC RETICULUM	163	5.49	15	0.000378	0.0147
RENAL CELL CARCINOMA	68	2.29	9	0.000421	0.0147
ERBB SIGNALING PATHWAY	84	2.83	10	0.000486	0.0148
VASOPRESSIN-REGULATED WATER REABSORPTION	43	1.45	7	0.000517	0.0148
COLORECTAL CANCER	88	2.96	10	0.000706	0.0185
CHRONIC MYELOID LEUKEMIA	76	2.56	9	0.000962	0.023
PATHWAYS IN CANCER	535	18	32	0.00103	0.023
PROSTATE CANCER	97	3.26	10	0.00151	0.0316
PHOSPHATIDYLINOSITOL SIGNALING SYSTEM	98	3.3	10	0.00163	0.032
LONG-TERM POTENTIATION	67	2.25	8	0.00174	0.0321
NEUROTROPHIN SIGNALING PATHWAY	121	4.07	11	0.00247	0.0431
REGULATION OF ACTIN CYTOSKELETON	217	7.3	16	0.00264	0.0437
GNRH SIGNALING PATHWAY	90	3.03	9	0.00317	0.0497
GLIOMA	75	2.52	8	0.00356	0.0533
THYROID HORMONE SIGNALING PATHWAY	115	3.87	10	0.00527	0.0716
EGFR TYROSINE KINASE INHIBITOR RESISTANCE	80	2.69	8	0.0053	0.0716
CENTRAL CARBON METABOLISM IN CANCER	64	2.15	7	0.00547	0.0716
MTOR SIGNALING PATHWAY	155	5.22	12	0.00599	0.0743
METABOLIC PATHWAYS	1490	50.2	67	0.00615	0.0743

PEROXISOME	84	2.83	8	0.0071	0.0826
WNT SIGNALING PATHWAY	160	5.38	12	0.00766	0.0859
ACUTE MYELOID LEUKEMIA	69	2.32	7	0.00824	0.0892
HIF-1 SIGNALING PATHWAY	105	3.53	9	0.00872	0.0895
HEPATITIS B	163	5.49	12	0.00883	0.0895
ENDOCYTOSIS	270	9.09	17	0.00959	0.0941
ADHERENS JUNCTION	72	2.42	7	0.0103	0.0955
B CELL RECEPTOR SIGNALING PATHWAY	72	2.42	7	0.0103	0.0955
AUTOPHAGY - ANIMAL	130	4.38	10	0.0121	0.109
ENDOCRINE RESISTANCE	93	3.13	8	0.0128	0.112
HIPPO SIGNALING PATHWAY	154	5.18	11	0.0147	0.122
ESTROGEN SIGNALING PATHWAY	134	4.51	10	0.0148	0.122
SALMONELLA INFECTION	78	2.63	7	0.0156	0.126
LONG-TERM DEPRESSION	61	2.05	6	0.0162	0.127
CHOLINE METABOLISM IN CANCER	99	3.33	8	0.0182	0.138
PROTEASOME	46	1.55	5	0.0185	0.138
INSULIN SIGNALING PATHWAY	140	4.71	10	0.0196	0.143
T CELL RECEPTOR SIGNALING PATHWAY	101	3.4	8	0.0202	0.144
MAPK SIGNALING PATHWAY	294	9.89	17	0.0207	0.145
CELL CYCLE	123	4.14	9	0.0227	0.153
NON-SMALL CELL LUNG CANCER	66	2.22	6	0.0231	0.153
SPHINGOLIPID SIGNALING PATHWAY	124	4.17	9	0.0238	0.153
STEROID BIOSYNTHESIS	19	0.639	3	0.0245	0.153
2-OXOCARBOXYLIC ACID METABOLISM	19	0.639	3	0.0245	0.153
RNA TRANSPORT	167	5.62	11	0.0253	0.153
GAP JUNCTION	86	2.89	7	0.0254	0.153
FC EPSILON RI SIGNALING PATHWAY	68	2.29	6	0.0264	0.156
FC GAMMA R-MEDIATED PHAGOCYTOSIS	87	2.93	7	0.0269	0.156
FRUCTOSE AND MANNOSE METABOLISM	35	1.18	4	0.029	0.165
PI3K-AKT SIGNALING PATHWAY	358	12	19	0.0329	0.184
OTHER TYPES OF O-GLYCAN BIOSYNTHESIS	22	0.74	3	0.0362	0.199
BACTERIAL INVASION OF EPITHELIAL CELLS	74	2.49	6	0.0379	0.203

HTLV-I INFECTION	246	8.28	14	0.0381	0.203
PANCREATIC CANCER	75	2.52	6	0.04	0.21
SIGNALING PATHWAYS REGULATING PLURIPOTENCY OF STEM CELLS	137	4.61	9	0.0414	0.213
PROTEOGLYCANS IN CANCER	204	6.87	12	0.0426	0.216
HEPATITIS C	160	5.38	10	0.0435	0.217
APELIN SIGNALING PATHWAY	139	4.68	9	0.0447	0.217
VEGF SIGNALING PATHWAY	58	1.95	5	0.0448	0.217
BLADDER CANCER	41	1.38	4	0.048	0.228
RAP1 SIGNALING PATHWAY	209	7.03	12	0.0496	0.232

Table S4. Pathway network enrichment analysis for DEU genes at TSK KO SVZ tissue. Pathway enrichments were estimated for KEGG. FDR = False discovery rate.

PATHWAY	TOTAL	EXPECTED	HITS	P.VALUE	FDR
SPLICEOSOME	133	0.25	3	0.00181	0.568
RIBOFLAVIN METABOLISM	8	0.0151	1	0.015	1
THIAMINE METABOLISM	15	0.0282	1	0.0279	1
NITROGEN METABOLISM	17	0.032	1	0.0316	1
ARGININE BIOSYNTHESIS	19	0.0358	1	0.0352	1
RIBOSOME	175	0.329	2	0.0419	1
GLYOXYLATE AND DICARBOXYLATE METABOLISM	31	0.0584	1	0.0568	1
BASE EXCISION REPAIR	35	0.0659	1	0.064	1
ALANINE, ASPARTATE AND GLUTAMATE METABOLISM	38	0.0715	1	0.0693	1
ARGININE AND PROLINE METABOLISM	50	0.0941	1	0.0902	1

Table S5. Estimation of protein-protein interaction network for DEU genes in TSK KO SVZ tissue.

LABEL	ID	DEGREE	BETWEENNESS
RPS26	27370	92	9724
RPL15	66480	92	2808
PAIP1	218693	20	9379
HNRNPA1	15382	20	4689
DCP1B	319618	13	2010
ATXN2	20239	12	3785
SRSF6	67996	10	929.5
SRSF5	20384	8	584.5
SRSF1	110809	3	1793.38
EIF3G	53356	2	7448
HNRNPD	11991	2	4433
PABPC1	18458	2	3725
DDX6	13209	2	2093
U2AF2	22185	2	0.38
NCBP2	68092	2	0.38
SRSF9	108014	2	0.38
SRSF2	20382	2	0.38
SRSF11	69207	2	0.38
SRPK1	20815	2	0.38
SRSF3	20383	2	0.38
RPL13	270106	2	0
RPS11	27207	2	0
RPL8	26961	2	0
RPS5	20103	2	0
RPS9	76846	2	0
RPLP1	56040	2	0
RPS18	20084	2	0
RPL10	110954	2	0
RPL19	19921	2	0
RACK1	14694	2	0
RPL24	68193	2	0
RPS14	20044	2	0
RPS3A1	20091	2	0
RPL6	19988	2	0
RPL28	19943	2	0
RPS3	27050	2	0
RPS4X	20102	2	0
RPL4	67891	2	0
RPSA	16785	2	0
RPS29	20090	2	0
RPL21	19933	2	0
EEF1A1	13627	2	0

RPL22L1	68028	2	0
RPS27L	67941	2	0
EEF2	13629	2	0
RPL37	67281	2	0
RPL10A	19896	2	0
RPS23	66475	2	0
RPL18A	76808	2	0
RPS21	66481	2	0
RPL37A	19981	2	0
RPS27RT	100043813	2	0
RPS15	20054	2	0
RPL7	19989	2	0
RPL26	19941	2	0
RPS12	20042	2	0
RPS7	20115	2	0
RPL17	319195	2	0
RPS25	75617	2	0
RPL36	54217	2	0
RPS17	20068	2	0
RPL35	66489	2	0
RPL29	19944	2	0
RPL3	27367	2	0
RPL32	19951	2	0
RPL5	100503670	2	0
RPLP2	67186	2	0
RPLP0	11837	2	0
RPL34	68436	2	0
UBA52	22186	2	0
RPL27	19942	2	0
RPS2	16898	2	0
RPL23	65019	2	0
RPL23A	268449	2	0
RPL11	67025	2	0
RPS8	20116	2	0
RPS6	20104	2	0
RPS27A	78294	2	0
RPL7A	27176	2	0
RPL38	67671	2	0
RPS16	20055	2	0
RPS19	20085	2	0
RPL30	19946	2	0
RPL36AL	66483	2	0
TPT1	22070	2	0
RPL36A	19982	2	0
RPL9	20005	2	0
RPS28	54127	2	0

RPS10	67097	2	0
RPL35A	57808	2	0
RPL39	67248	2	0
RPL13A	22121	2	0
EEF1B2	55949	2	0
RPL12	269261	2	0
RPL22	19934	2	0
RPS15A	267019	2	0
RPS20	67427	2	0
RPL27A	26451	2	0
RPS24	20088	2	0
RPS13	68052	2	0
RPL3L	66211	2	0
RPL14	67115	2	0
RPS27	57294	2	0
FAU	14109	2	0
RPL31	114641	2	0
LSM2	27756	1	0
DDX5	13207	1	0
LSM12	268490	1	0
ATXN3	110616	1	0
DCP1A	75901	1	0
EIF3H	68135	1	0
EIF4A2	13682	1	0
DCP2	70640	1	0
EIF3A	13669	1	0
PA2G4	18813	1	0
EIF4E	13684	1	0
WDR31	71354	1	0
SH3GL2	20404	1	0
EIF3F	66085	1	0
LSM4	50783	1	0
XRN1	24127	1	0
ATXN2L	233871	1	0
CNOT3	232791	1	0
EDC4	234699	1	0
HNRNPK	15387	1	0
LSM1	67207	1	0
RPL39L	68172	1	0
LSM3	67678	1	0
EIF5A	276770	1	0
HNRNPU	51810	1	0
PABPC5	93728	1	0
EDC3	353190	1	0
LSM6	78651	1	0
RBFOX1	268859	1	0

PABPC6	67543	1	0
PCBP1	23983	1	0
DMRT2	226049	1	0
PATL1	225929	1	0
CNOT10	78893	1	0
KHDRBS1	20218	1	0
PABPC2	18459	1	0
TUT7	214290	1	0
PCBP2	18521	1	0
HNRNPH1	59013	1	0
TARDBP	230908	1	0
CNOT9	58184	1	0
FMR1	14265	1	0
RPL7L1	66229	1	0
TIA1	21841	1	0
EEF1G	67160	1	0
AGO1	236511	1	0
TUT4	230594	1	0
CNOT1	234594	1	0
PABPC1L	381404	1	0
EIF3I	54709	1	0
RPL10L	238217	1	0
FUS	233908	1	0
TNPO1	238799	1	0
TNKS1BP1	228140	1	0
HNRNPC	15381	1	0
ZRSR2	22184	1	0
TSR2	69499	1	0
HNRNPH2	56258	1	0
HNRNPA2B1	53379	1	0
HNRNPM	76936	1	0
TRA2B	20462	1	0
PTBP1	19205	1	0
PABPC4L	241989	1	0
LSM5	66373	1	0
EIF4A1	13681	1	0
EIF4B	75705	1	0
DROSHA	14000	1	0
HNRNPF	98758	1	0
HNRNPL	15388	1	0
HNRNPR	74326	1	0

Table S6. Pathway network enrichment analysis for DEU genes in TSK KO muscle tissue. Pathway enrichments were estimated for KEGG. FDR = False discovery rate.

PATHWAY	TOTAL	EXPECTED	HITS	P.VALUE	FDR
PENTOSE PHOSPHATE PATHWAY	32	0.0527	3	1.72E-05	0.00539
GLYCOLYSIS / GLUCONEOGENESIS	67	0.11	3	0.00016	0.0252
PLATELET ACTIVATION	125	0.206	3	0.001	0.101
STARCH AND SUCROSE METABOLISM	33	0.0544	2	0.00129	0.101
TIGHT JUNCTION	167	0.275	3	0.00231	0.145
AMINO SUGAR AND NUCLEOTIDE SUGAR METABOLISM	49	0.0807	2	0.00284	0.148
FOCAL ADHESION	199	0.328	3	0.00381	0.171
BIOSYNTHESIS OF AMINO ACIDS	78	0.129	2	0.00705	0.246
CARDIAC MUSCLE CONTRACTION	78	0.129	2	0.00705	0.246
HYPERTROPHIC CARDIOMYOPATHY (HCM)	86	0.142	2	0.00851	0.265
DILATED CARDIOMYOPATHY	90	0.148	2	0.00929	0.265
HIF-1 SIGNALING PATHWAY	105	0.173	2	0.0125	0.327
LEUKOCYTE TRANSENDOTHELIAL MIGRATION	115	0.189	2	0.0149	0.359
CARBON METABOLISM	120	0.198	2	0.0161	0.361
ADRENERGIC SIGNALING IN CARDIOMYOCYTES	148	0.244	2	0.0239	0.5
CGMP-PKG SIGNALING PATHWAY	172	0.283	2	0.0316	0.62
AXON GUIDANCE	180	0.297	2	0.0344	0.635
HIPPO SIGNALING PATHWAY -MULTIPLE SPECIES	26	0.0428	1	0.042	0.733
REGULATION OF ACTIN CYTOSKELETON	217	0.357	2	0.0483	0.798

Table S7. Key pathway associated DEGs in SVZ GFAP⁺ cells. Associated DEGs were inferred from KEGG database. Blue and orange highlights indicate increase and decrease of the expression respectively.

Pathway	Gene	Log2FC
**Splisosome**	Hspa2	2.02919107
	Hspa8	0.9068062
	Ncbp2	0.81017099
	Srsf5	0.4635995
	Prpf6	0.42872292
	Pcbp1	0.37265418
	U2af2	0.21817372
	Ddx23	0.20444152
	Puf60	0.19802751
	Dhx16	0.1745822
	Prpf40a	-0.1374434
	Snrnp70	-0.1793712
	Ncbp1	-0.1925065
	Ddx39b	-0.2682819
	Ddx5	-0.3048301
	Srsf4	-0.311985
	Rbm25	-0.3227002
	Hnrnpc	-0.3328301
	Hnrnpm	-0.3443135
	Hnrnpa3	-0.3443135
	Tra2b	-0.3538379
	Srsf3	-0.3870482
	Tcerg1	-0.3882383
	Ppie	-0.4189508
	Srsf10	-0.4318186
	Dhx15	-0.4497596
	Prpf18	-0.5429913
	Xab2	-0.6647736
**Wnt Signaling**	Ruvbl1	2.000265
	Jun	0.8140987
	Gpc4	0.700756
	Ctbp2	0.6785677
	Fzd8	0.6548163
	Dvl2	0.6137053
	Ccnd3	0.442295
	Daam2	0.425649
	Tbl1xr1	0.4195416
	Fzd3	0.4080511
	Prkaca	0.3800168
	Smad3	0.3509944

	Plcb3	0.3211464
	Cacybp	0.2549645
	Rbx1	0.1646927
	Ep300	-0.179331
	Chd8	-0.2132859
	Csnk1a1	-0.2682395
	Daam1	-0.2753009
	Map3k7	-0.35086
	Smad4	-0.3711098
	Axin2	-0.3944007
	Apc	-0.4052626
	Siah1a	-0.4170756
	Camk2g	-0.7652321
**Cell Cycle**	Ccna1	1.796805
	Mad1l1	0.7005939
	Cdc7	0.489599
	Bub1	0.4785446
	Ccnd3	0.442295
	Ywhah	0.360927
	Smad3	0.3509944
	Abl1	0.2285213
	Ywhae	0.2272228
	Rbx1	0.1646927
	Anapc4	-0.1433438
	Ep300	-0.179331
	Smc3	-0.2487972
	Anapc2	-0.2670151
	Anapc7	-0.3047196
	Smad4	-0.3711098
	Cdk2	-0.3914618
	Orc6	-0.3997562
	Zbtb17	-0.4032115
	Rbl2	-0.4198816
	Orc4	-0.4217027
	Orc3	-0.5204759
**Circadian Rhythm**	Prkag1	0.65078599
	Prkaa1	0.32770806
	Rbx1	0.16469273
	Prkab1	-0.3504476
	Cry2	-0.3554909
	Clock	-0.4366392
	Per2	-0.5538372
	Nr1d1	-0.7151754
	Bhlhe40	-0.7415495
	Per1	-0.9516663

**SHH Pathway**	Gli2	0.84200869
	Boc	0.77170314
	Megf8	0.41943073
	Prkaca	0.38001676
	Smo	0.27456315
	Arrb1	-0.1371539
	Csnk1a1	-0.2682395
	Smurf2	-0.3278283
	Cdon	-0.6428056
	Gas1	-4.306482
**mTOR Pathway**	Nprl2	1.59471705
	Prr5l	1.02614928
	Tti1	0.7294427
	Fzd8	0.65481629
	Mapkap1	0.65076256
	Dvl2	0.61370528
	Stradb	0.51206441
	Depdc5	0.46215876
	Mtor	0.42543042
	Ikbkb	0.419569
	Fzd3	0.40805105
	Ulk2	0.38285592
	Prkaa1	0.32770806
	Rps6ka6	0.31000086
	Hras	0.30710054
	Map2k1	0.29966344
	Sos1	0.29507647
	Rps6ka3	0.25207776
	Atp6v1e1	-0.1931713
	Mapk1	-0.2433967
	Kras	-0.2633941
	Raf1	-0.2734757
	Igf1r	-0.2765847
	Chuk	-0.2810746
	Cab39	-0.3382156
	Atp6v1b2	-0.3691205
	Atp6v1c1	-0.3906418
	Rictor	-0.3918546
	Slc7a5	-0.7081074

Data file S1. Estimation of significant DEGs in SVZ $GFAP^+$ cells by Ballgawn. Significant ($p < 0.05$) differentially expressed transcripts with gene assignment. fc = fold change, pval = p value, and qval = q value for the transcript. Empty rows in the gene name column denote novel transcripts.

Data file S2. Significant DEGs that have DEUs in $GFAP^+$ cells. Common genes in the Ballgawn significant ($p < 0.05$) DEGs and DEXseq significant (FDR <0.1) DEU genes. Blue and orange highlights indicate increase and decrease of expression respectively.

Data file S3. Significant DEU genes with chromosomal location and number of exon changing events in SVZ $GFAP^+$ cells. DEXseq output for genes with significant (FDR < 0.1) differential exon usage. chr = chromosome.

Data file S4. Significant DEU genes with chromosomal location and number of exon changing events in SVZ tissue. DEXseq output for genes with significant (FDR < 0.05) differential exon usage. chr = chromosome.

Data file S5. Significant DEU genes with chromosomal location and number of exon changing events in muscle tissue. DEXseq output for genes with significant (FDR < 0.05) differential exon usage. chr = chromosome.

Data file S6. String-based protein-protein interaction (PPI) analysis for TSK and spliceosome associated factors that have DEU. Estimation of confidence score between nodes(proteins). Minimum interaction score: 0.4
